# Developmental vascular remodeling defects and postnatal kidney failure in mice lacking Gpr116 (Adgrf5) and Eltd1 (Adgrl4)

**DOI:** 10.1371/journal.pone.0183166

**Published:** 2017-08-14

**Authors:** Shun Lu, Shuya Liu, Astrid Wietelmann, Baktybek Kojonazarov, Ann Atzberger, Cong Tang, Ralph Theo Schermuly, Hermann-Josef Gröne, Stefan Offermanns

**Affiliations:** 1 Department of Pharmacology, Max Planck Institute for Heart and Lung Research, Bad Nauheim, Germany; 2 Scientific Service Group Nuclear Magnetic Resonance Imaging, Max Planck Institute for Heart and Lung Research, Bad Nauheim, Germany; 3 Department of Internal Medicine, Justus-Liebig-University Giessen, Universities of Giessen and Marburg Lung Center (UGMLC), Member of the German Center for Lung Research (DZL), Giessen, Germany; 4 Flow Cytometry Service Facility, Max Planck Institute for Heart and Lung Research, Bad Nauheim, Germany; 5 Department of Cellular and Molecular Pathology, German Cancer Research Center (DKFZ), Heidelberg, Germany; 6 Medical Faculty, J.W. Goethe University Frankfurt, Frankfurt, Germany; Ludwig-Maximilians-Universitat Munchen, GERMANY

## Abstract

GPR116 (ADGRF5) and ELTD1 (ADGRL4) belong to different subfamilies of the adhesion G-protein-coupled receptor group but are both expressed in endothelial cells. We therefore analyzed their functions in mice lacking these receptors. While loss of GPR116 or ELTD1 alone had no obvious effect on cardiovascular or kidney function, mice lacking both, GPR116 and ELTD1, showed malformations of the aortic arch arteries and the cardiac outflow tract leading to perinatal lethality in about 50% of the mutants. In addition to cardiovascular malformations, surviving mice developed renal thrombotic microangiopathy as well as hemolysis and splenomegaly, and their lifespan was significantly reduced. Loss of GPR116 and ELTD1 specifically in endothelial cells or neural crest-derived cells did not recapitulate any of the phenotypes observed in GPR116-ELTD1 double deficient mice, indicating that loss of GPR116 and ELTD1 expressed by other cells accounts for the observed cardiovascular and renal defects.

## Introduction

G-protein-coupled receptors (GPCRs) constitute the largest receptor family in eukaryotes [1]. They consist of five families in mammals, among which the adhesion GPCR family is the second largest with 33 members in humans and 31 members in mice [2]. Many adhesion GPCRs have long N-terminal extracellular regions that contain various domains with adhesive functions. The extracellular N-terminal part of the protein can be autoproteolytically cleaved at the GPCR proteolysis site (GPS) motif, which is highly conserved and located close to the first transmembrane helix [3, 4]. The GPS motif is part of a much larger domain, the GPCR autoproteolysis inducing (GAIN) domain, which is functionally necessary and sufficient for autoproteolysis [5]. After autoproteolysis, the N-terminal and C-terminal parts of the receptor remain non-covalently attached to each other, but this interaction can be disrupted by binding of extracellular proteins or by physical forces [3, 6]. It is still unclear whether the N-terminal part of the receptor exists then as a soluble fragment and to which degree it affects the function of the C-terminal part containing the seven transmembrane (7TM) domains in different adhesion GPCR subtypes. In some cases, the isolated 7TM domain-containing part has been shown to possess constitutive activity [7], and in some adhesion GPCRs the neo amino terminus may function as a tethered agonist [8, 9].

ADGRL4 (ELTD1) is a member of the latrophilin-subfamily based on its 7TM sequence homology [3]. The epidermal growth factor (EGF) and calcium-binding EGF domains in the extracellular part of ELTD1 have homology with extracellular domains of “EGF-7TM” family members, including EMR1-4 and CD97. ELTD1, whose sequence is highly conserved across vertebrates [10], was described as a receptor upregulated in the adult heart [11]. Later on, evidence was provided that Eltd1 counteracts pressure overload-induced myocardial hypertrophy [12] and regulates tumor angiogenesis [13]. ADGRF5 (GPR116) is grouped into adhesion GPCR subfamily VI with 4 related receptors [2, 3]. It has a well-established role in lung surfactant homeostasis, and lack of GPR116 leads to progressive accumulation of surfactant lipids and proteins in the alveolar space of mice [14–17]. A selective deletion of GPR116 in mouse adipose tissue was reported to cause glucose intolerance and insulin resistance [18]. More recently, loss of Gpr116 has also been reported to result in a subtle vascular phenotype including a leakiness of the blood-brain-barrier and a reduced pathological response in a model of oxygen-induced retinopathy [17].

Although Eltd1 and Gpr116 belong to different subfamilies of adhesion GPCRs, they have in common to be highly expressed in the microvascular endothelium [19]. Another study also reported the expression of Gpr116 in endothelial cells in early mouse embryos [20], and endothelial ELTD1 was suggested to be highly associated with tumor angiogenesis [13]. In the context of angiogenesis, ELTD1 was demonstrated to be up-regulated by VEGF and down regulated by DLL4 [21]. Based on the coexpression of both receptors in endothelial and other cells, we sought to explore the role of GPR116 and ELTD1 using global and conditional knockout (KO) mice.

## Materials and methods

### Animals

Transgenic mice expressing mCherry under the control of the *Gpr116* promoter (*Gpr116*-mCherry) were generated using the BAC clone RP24-510M8 (CHORI, CA, USA) from mouse chromosome 17 containing the *Gpr116* gene. The coding sequence of the *Gpr116* gene on the BAC was replaced by a cassette carrying the mCherry cDNA followed by a polyadenylation signal and an FRT-flanked ampicillin resistant gene (β-lactamase) using Red/ET recombination kit (Gene Bridges). Correct targeting was verified by restriction digests and DNA sequencing. After Flp-mediated excision of the ampicillin resistant gene and linearization, the recombined BACs were injected into pronuclei of FVB/N oocytes. Transgenic offspring was genotyped for BAC insertion by genomic PCRs. Two different founders were used to generate the *Gpr116* reporter line in which mCherry expression was determined by fluorescence microscopy of 8–12 μm cryosections of various tissues. Both lines generated with the same transgene showed a comparable expression pattern for mCherry. Animals were kept on a C57BL/6 background. For genotyping by PCR the following primers were used: forward: 5’-CTTCATCATGTCCACAGAACC-3’; reversed: 5’-AGGATGTCCCAGGCGAAGG-3’; PCR products of 502 basepairs (bp) indicated the transgenic allele. Eltd1 Lacz knockin line was obtained from Knockout Mouse Project (www.komp.org) with Project ID: VG12398.

Mice with null or floxed alleles of *Gpr116* and *Eltd1* were generated after gene targeting in embryonic stem (ES) cells. In V6.5 (C57BL/6 x 129S4/SvJae) ES cells (Novus Biologicals), exon 8 of *Gpr116* or exon 5 of *Eltd1* was replaced by a cassette carrying the neomycin resistance gene (flanked by FRT recombination sites) via homologous recombination. Correct targeting was verified by Southern blotting and PCR. Highly chimeric males obtained from targeted ES cell clone injection were bred onto C57BL/6 background. F1 generation mice carrying targeted allele were mated either with flp recombinase expressing mice [22] resulting in removal of the neo cassette, to generate *Gpr116*- or *Eltd1-* floxed mice, or were mated with EIIa-Cre mice [23] to generate a null allele of *Gpr116* or *Eltd1* by recombining loxP sites in the early embryonic stage. Deletion of the targeted exon was confirmed by both quantitative RT-PCR (qPCR) and RT-PCR. Animals were back-crossed with C57BL/6N mice for at least 8 generations. *Gpr116/Eltd1* double knockout (*Gpr116*^-/-^*;Eltd1*^-/-^) mice were generated by crossing *Gpr116*^-/-^ animals with *Eltd1*^-/-^ animals. To generate conditional double knockout mice, *Gpr116*^flox/flox^;*Eltd1*^flox/flox^ mice were crossed with vascular endothelial cadherin (VE-Cad)-Cre mice [24] or Pax3-Cre mice [25].

Mice were housed under a 12-h light–dark cycle with free access to food and water and under specific pathogen-free conditions. All animal procedures were approved by the Institutional Animal Care and Use Committee of the Regierungspräsidia Karlsruhe and Darmstadt.

### Genotyping of mice

For genotyping the *Gpr116* null and floxed allele (S3 Fig), the following primers were used: primer (P) 1: 5’-CGTGGGCTATCATGTAGGGTCC-3’; P2: 5’-CATCCC-GAGATCCTGTCTGCCTAT-3’; P3: 5’-GGGTAGGCATGATGTGATGGTATT-3’; P4: 5’-TTTGTTTCCCTGAGTCTGGTTCAT-3’. For genotyping the *Eltd1* null and floxed allele (S4 Fig), the following primers were used: P1: 5’-CGGGGAAGTCACAGTTCACACC-3’; P2: 5’-GTAGCTCACATTCATTCTTTCTTCC-3’; P3: 5’-CAGCCATGTATGACTTACGTTGCAGA-3’; P4: 5’- ATGTGGTACTGGA-TCTGAGCCTATGT-3’. Gene deletion was further confirmed by reverse-transcription PCR (RT-PCR). Hearts, lungs and kidneys were prepared from sacrificed animals. Total mRNA was extracted using the RNAeasy kit (Qiagen) and cDNA synthesis was performed by using the Transcriptor First Strand cDNA Synthesis Kit (Roche Applied Science). Following primers were used in RT-PCR: *Gpr116* P5: 5’-CGAGCGGGTTACAGAACTTTAC-3’; *Gpr116* P6: 5’-CTCCATAGTACCAAG-ACGTGTTG-3’; *Eltd1* P5: 5’-CTTCTGTCTGCGGTGATCATGC-3’ and *Eltd1* P6: 5’- GGGTGGCCTAGTAGTGAGGATG-3’ (S3 and S4 Figs).

### Immunofluorescence microscopy

8 μm cryo-sections were fixed in 4% PFA for 10 min, washed three times in PBS, incubated for 30 min with 5% BSA in PBS + 0.1% Triton for blocking and permeabilization, and sections were then incubated overnight at 4°C with primary antibodies (anti-CD31, 1:200, Cat.550274, BD Bioscience; anti-Podocin, 1:600, Cat.P0372, Sigma-Aldrich; anti-Desmin, 1:400, Cat.D10033, Sigma-Aldrich; anti-Prosurfactant Protein C, 1:300, Cat.AB3786, Millipore). Slices were washed three times in PBS and then incubated for 1 h at room temperature with secondary antibodies (1:200, Life Technologies) and DAPI (4',6-diamidino-2-phenylindole) (1:10000, Invitrogen). After washing three times in PBS, slices were mounted and analyzed using the Leica TCS SP5 or Zeiss Axio Observer.Z1 microscope.

### Histochemistry and Electron Microscopy (EM)

X-ßGal-staining: 8-μm tissue cryo-sections were fixied in 0.1 M PBS, pH 7.3, 5 mM EGTA, 2 mM MgCl_2_, 0.2% Glutardialdehyde for 5 min at RT, washed three time in ice old wash buffer (0.1 M PBS, pH 7.3, 5 mM EGTA, 2 mM MgCl_2_, 0.01% sodium deoxycholate, 0.02% NP-40), and sections were then incubated 8 hours at 37°C in X-ßGal reaction buffer (10 mM potassium ferrocyanide, 10 mM potassium ferricyanide, 0.5 mg/ml X-ßGal reagent (Cat.2315, Carl Roth) in wash buffer), washed two times with PBS and followed by eosin counterstaining. SPiDER-ßGal staining: 8-μm cryo-sections were fixed in 0.2% PFA for 10 min at RT, washed three times in PBS, incubated for 1 hour with 1 μmol/l SPiDER-ßGal (SG02-10, Dojindo EU GmbH), 0.1% Triton in PBS at 37°C. Sections were then blocked and stained with antibodies described above. Under these conditions, no activity was observed in non-transgenic animals. Periodic acid-Schiff (PAS) (Cat.395B, Sigma-Aldrich)-staining was performed on 6-μm kidney paraffin sections. Hematoxylin & eosin (HE)-staining was performed on 10-μm embryo paraffin sections. Goldner Trichrome staining as well as transmission electron microscopy were performed as described [26].

### Urine analysis

Mouse urine samples were collected and centrifuged for 5 min at 900 x g to remove cell debris. 45 μl of urine were mixed with 15 μl Laemmli (4x) and incubated for 5 min at 99°C, and 10 μl of the mixture was loaded onto an 8% SDS-PAGE. Bovine serum albumin (BSA) (New England BioLabs) was used as control. After protein separation, the gel was fixed in a fixation buffer (25% (v/v) isopropanol and 10% (v/v) acetic acid) for 30 min, subsequently stained in Coomassie staining buffer (0.2% (w/v) Coomassie Brilliant Blue R-250 (Cat. 27816, Sigma-Aldrich), 40% methanol and 10% acetic acid) for 1 h and finally in destaining buffer (40% (v/v) methanol and 10% (v/v) acetic acid) for 2–4 h. The gel was scanned using HP Scanjet G4050.

### Magnetic resonance imaging (MRI)

All MRI experiments were performed on a 7.0T superconducting magnet (Bruker Biospin, Pharmascan, 70/16, Ettlingen, Germany) equipped with an actively shielded imaging gradient field of 760 mT/m operating at frequencies of 300.1 MHz [27]. Mice were measured under volatile isoflurane (1.5–2.0% in oxygen and air with a flow rate of 1.0 L/min) anesthesia; the body temperature was maintained 37°C by a thermostatically regulated water flow system during the entire imaging protocol.

### Vascular corrosion casting and Micro-CT imaging

Mice were deeply anesthetized with pentobarbital and perfused via the left ventricle, first with physiological 0.9% (w/v) NaCl containing heparin (25.000 U/l) followed by 4% (v/v) PFA at 4 ml/min. Both solutions were kept at 35°C. Immediately thereafter, the polymer PU4ii (vasQtec, Switzerland) was infused at the same rate. After resin curing (2 days, room temperature), soft tissue was macerated in 7.5% (w/v) KOH for 24 h at 0050°C and thereafter washed in tap water. Vascular casts were scanned using a Quantum GX cone-beam microCT imaging system (Rigaku Co., Tokyo, Japan). The system uses a CMOS flat-panel detector and a microfocus X-ray source mounted on a gantry with continuous rotation. 6,424 projection images were acquired over a 36-mm field of view (FOV) with the X-ray source set to 90 kV and 80 μA over a complete 360-degree rotation for a total scan time of 14 minutes. 2352 X 2944 detector elements were read and images were reconstructed using a modified filtered back-projection Feldkamp algorithm selecting 512 X 512 X 512 matrix size with a reconstructed isotropic voxel size of 20 μm. Image processing software Osirix 6.0 (Pixmeo, Switzerland) was used to generate 3D video and images.

### Blood analysis

Blood samples were collected in EDTA (10 mM final concentration). Blood smears were stained with Giemsa solution (Cat.32884, Sigma-Aldrich). Blood and plasma parameters were determined by IDEXX Laboratories, Ludwigsburg, Germany.

### Isolation of endothelial cells from the lung and real-time qPCR

Isolation of endothelial cells from the lung was reported previously [28]. Briefly, animals were killed, perfused with PBS and lungs were dissected, minced and enzymatically digested for 60 min while shaking at 37°C in a digestion mix containing collagenase II (2 mg/ml; Worthington), elastase-I (0.04 mg/ml; Sigma), DNase1 (5 U/ml; New England Biolabs) and dispase II (1.2 U/ml; Sigma). Cell suspensions were serially filtered through 70 and 40 μm cell strainers. Endothelial cells were enriched using the autoMACS (Milteny Biotec) after labelling with rat anti-mouse CD31-PE antibody (Cat.MCA2388PE, Serotec) and anti-PE magnetic microbeads (130-105-639, Milteny Biotec). Immune cells were labelled by rat anti-mouse CD45-FITC antibody (Cat.553079, BD Biosciences). Subsequently, using flow cytometric gating strategies to detect live single cells, the endothelial cells were identified and sorted based on CD31^+^/CD45^-^/DAPI^-^ expression. Endothelial cells (20000 cells per mouse) were directly sorted in lysis buffer for RNA isolation. RNA was prepared using RNAeasy Micro Kit (74004, Qiagen). First-strand cDNA was synthesized using ProtoScript II Reverse Transcriptase (M0368, New England BioLabs). The same reaction was performed without reverse transcriptase as non-RT control to test for genomic DNA contamination. The real-time qPCR was performed using the LightCycler 480 SYBR Green I Master System (Roche). Relative expression levels were obtained by normalization to *Actb* mRNA. Following real-time qPCR primers (intron-spanning) were used: *Gpr116* (forward): 5’-GGA CTA CAA CTC CTT CCA GGG-3’; *Gpr116* (reverse): 5’-CAG AGT CAC ATT GTC TCC CTC-3’; *Eltd1* (forward): 5’-GCC AAG AAA GCA TGA ATT CAA ATT GCC AC-3’; *Eltd1* (reverse): 5’-AGT TGT CTG TTC TGT GAG GGG TCC -3’; *Actb* (forward): 5’-CCC TAA GGC CAA CCG TGA AAA G-3’; *Actb* (reverse): 5’-CAG AGG CAT ACA GGG ACA GCA C-3’.

### Statistics

Data are presented as mean values ± SD. Statistical analyses were performed using GraphPad Prism 5 software. Paired *t* test was used in MRI study. For other studies unpaired *t* test was used to compare two groups. Differences were considered statistically significant at P ≤ 0.05.

## Results

### Expression of Gpr116 and Eltd1 in mice

**Fig 1:**
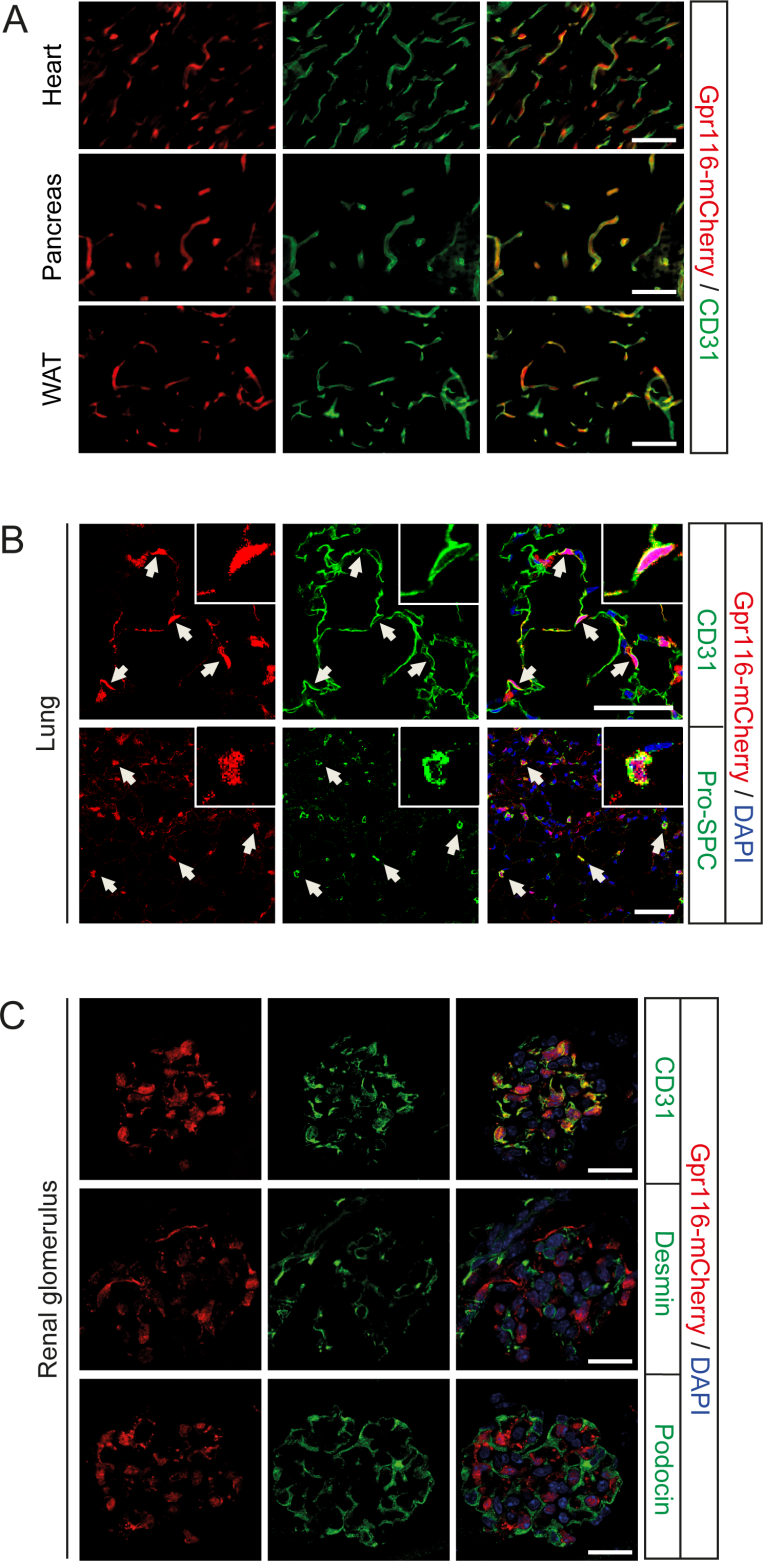
Expression of GPR116 in adult mice. (**A-C**) Representative fluorescent images (of 5 images per organ from 4 examined animals) of mCherry reporter mice, showing GPR116 expression at 8 weeks of age in the heart, pancreas and white adipose tissue (WAT) (**A**), in the lung (**B**) and renal glomeruli (**C**). Endothelial cells are stained with anti-CD31, alveolar epithelial type II cells are stained with anti-Pro-SPC, glomerular podocytes are stained with anti-podocin and glomerular mesangial cells are stained with anti-desmin antibodies. Nuclei are counterstained with DAPI. Scale bars: 50 μm (**A** and **B**); 20 μm (**C**).

**Fig 2:**
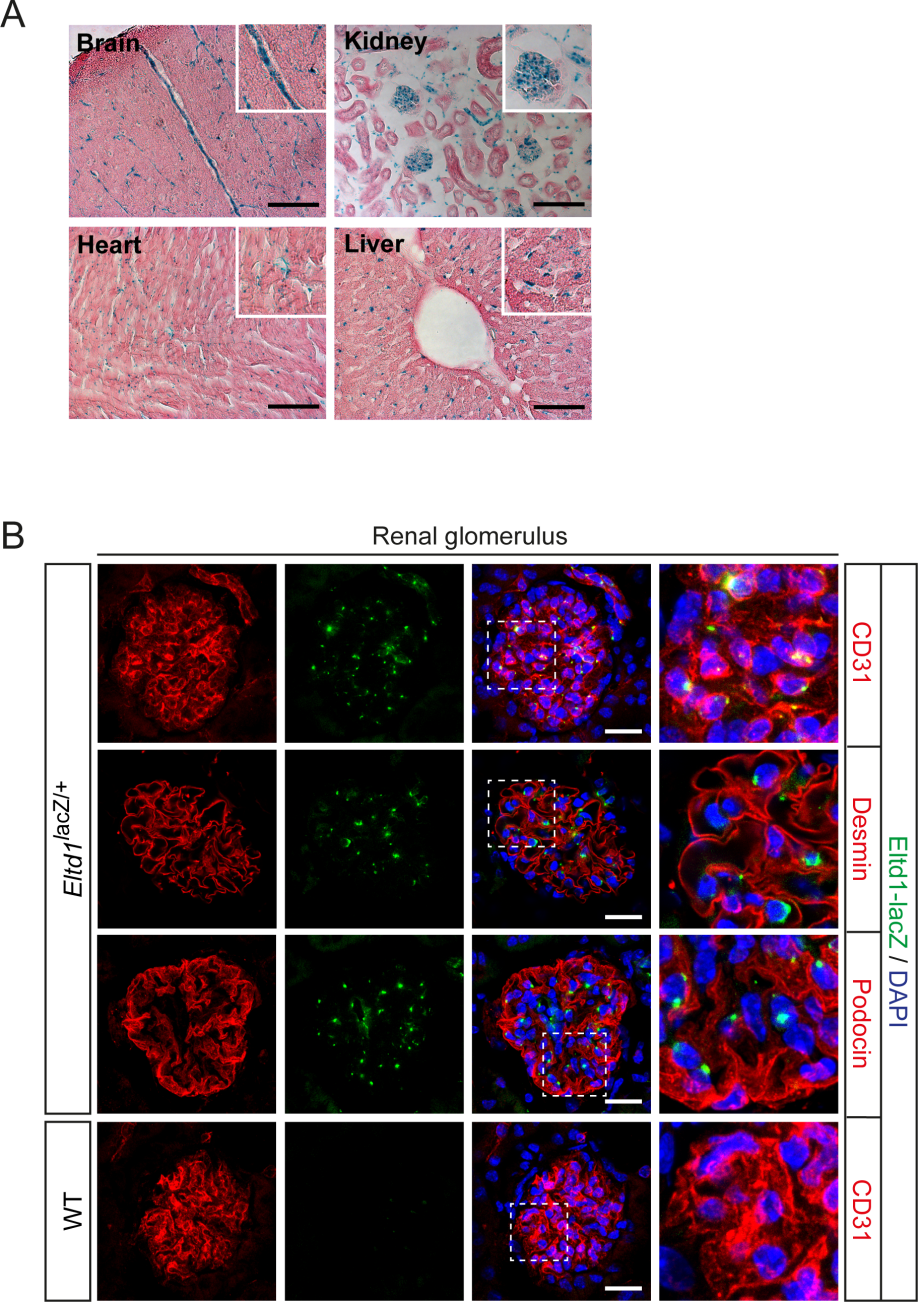
Expression of ELTD1 in adult mice. (**A**) Representative H&E staining of different organs from 8-week-old mice expressing ß-galactosidase under the control of the *Eltd1* promoter (*Eltd1*^*lacZ/+*^). Activity of ß-galactosidase is detected by X-gal blue staining. (**B**) Representative fluorescent images (of 9 images from 3 examined animals) of renal glomeruli in *Eltd1*^*lacZ/+*^ mice compared with WT mice. Activity of ß-galactosidase is detected by SPiDER-ßGal, endothelial cells are stained with anti-CD31, glomerular podocytes are stained with anti-podocin and glomerular mesangial cells are stained with anti-desmin antibodies. Nuclei are counterstained with DAPI. Scale bars: 200 μm (**A**); 50 μm (**B**).

To analyze the expression of *Gpr116* in mice, we generated a bacterial artificial chromosome (BAC)-based transgenic mouse line expressing mCherry under the control of the *Gpr116* promoter and enhancer elements (S1 Fig). Expression of Gpr116 co-localized with the endothelial cell marker CD31 and could be detected in microvessels in different organs including the heart, pancreas, adipose tissue (Fig 1A) and lung (Fig 1B). Gpr116 was also detected in alveolar epithelial type II cells of the lung (Fig 1B), which is consistent with previous studies showing an important role of Gpr116 in the alveolar surfactant homeostasis [14–17]. In the kidney, Gpr116 expression exclusively co-localized with the endothelial cell marker but not with desmin or podocin indicating mesangial cells or podocytes, respectively (Fig 1C). A knock-in mouse line expressing the lacZ gene under the control of the *Eltd1* promoter showed Eltd1 expression in microvessels of various organs including the brain, heart, kidney and liver (Fig 2A). In the kidney, Eltd1 expression exclusively co-localized with endothelial cells but not with mesangial cells or podocytes (Fig 2B).

**Fig 3:**
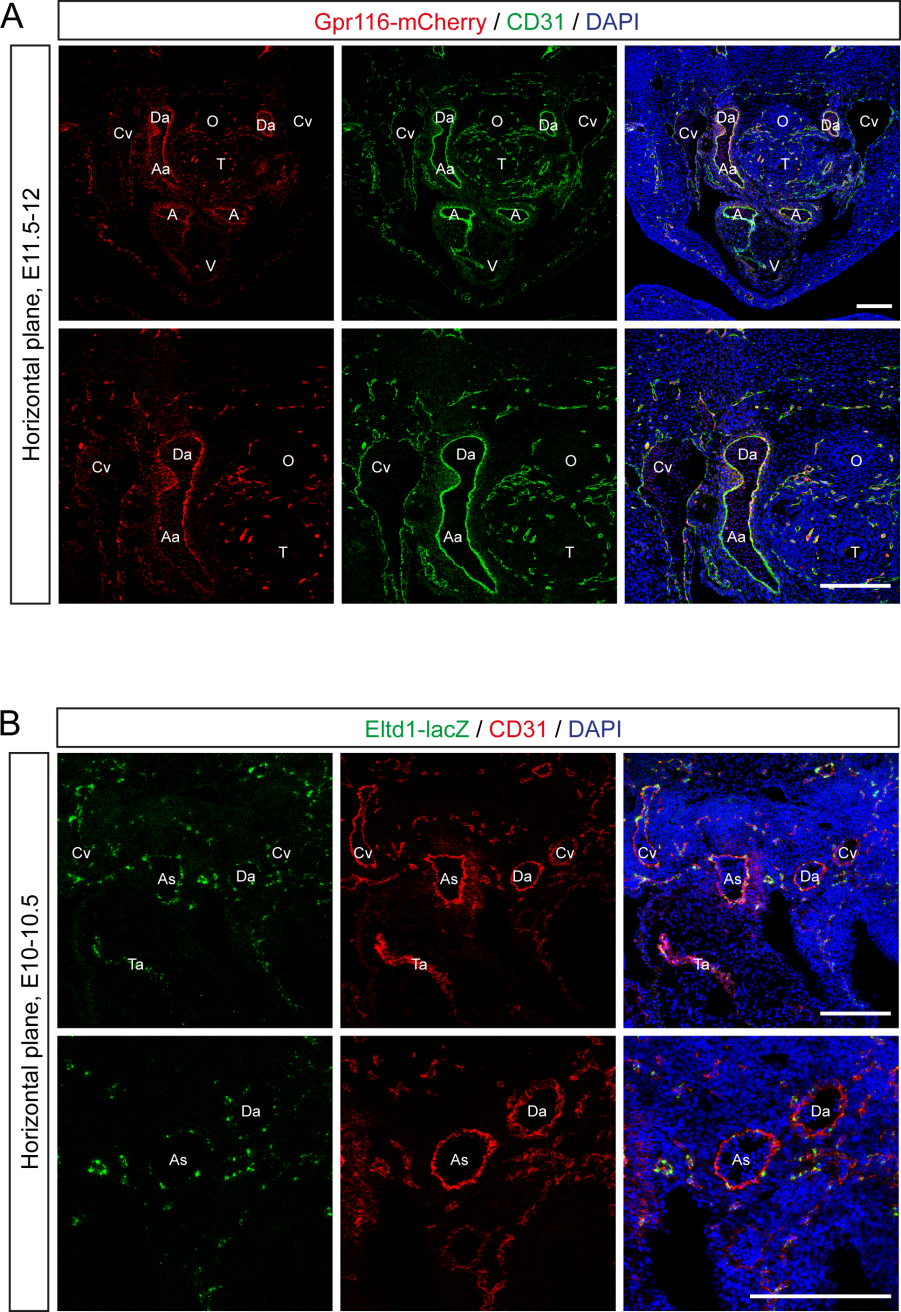
Expression of GPR116 and ELTD1 in mouse embryos. (**A**) Representative fluorescent images (of 18 images from 3 examined animals) of the heart and OFT area in Gpr116-mCherry reporter mice at embryonic day 11.5–12. Endothelial cells are stained with anti-CD31 antibody. (**B**) Representative fluorescent images (of 18 images from 3 examined animals) of the heart and OFT area in *Eltd1*^*lacZ/+*^ mice at embryonic day 10–10.5. Activity of ß-galactosidase is detected by SPiDER-ßGal, endothelial cells are stained with anti-CD31 antibody. (**A, B**) Nuclei are counterstained with DAPI. As, aortic sack; Da, dorsal aorta; Ta, truncus arteriosus; Cv, cardinal vein; Aa, arch arteries; A, atrium; V, ventricle; T, trachea; O, oesophagus. Scale bars: 200 μm.

Expression of Gpr116 and Eltd1 was also observed in the early stage of embryonal development (E10-12) and co-localized with endothelial cell marker CD31 in the developing heart, aortic arch arteries as well as other vessels (Fig 3A and 3B). At embryonic day 18.5, we also confirmed expression of Gpr116 and Eltd1 in endothelial cells in multiple organs including the heart, lung and skeletal muscle (S2 Fig).

### Perinatal lethality in mice lacking Gpr116 and Eltd1

**Fig 4:**
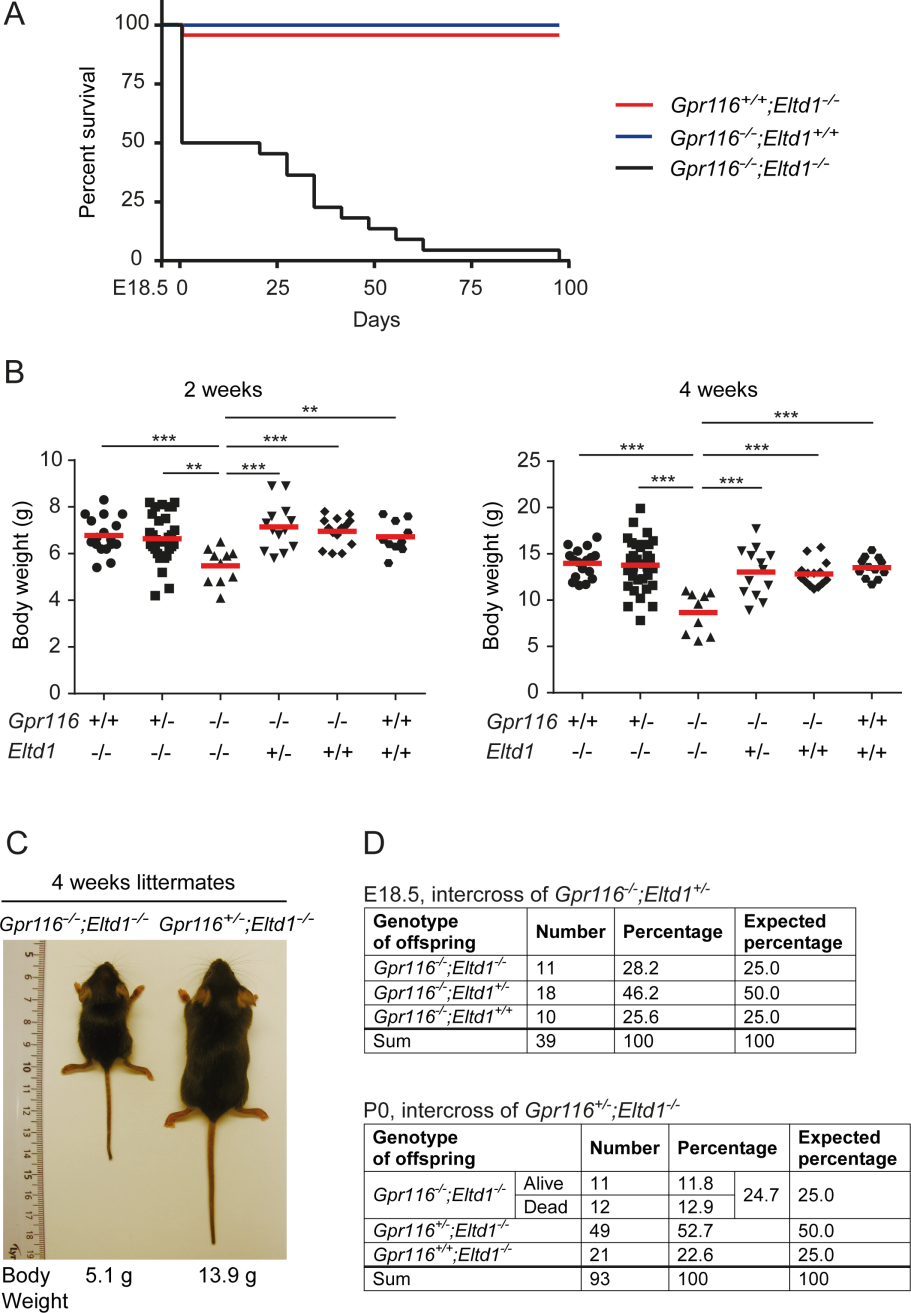
Life expectancy and body weight of GPR116-ELTD1 double deficient mice. (**A**) Kaplan-Meyer survival curve of *Gpr116*^*-/-*^*;Eltd1*^*-/-*^, *Gpr116*^*+/+*^*;Eltd1*^*-/-*^ and *Gpr116*^*-/-*^*;Eltd1*^*+/+*^ mice (n ≥ 11 per group). (**B**) Bodyweight of *Gpr116*^*-/-*^;*Eltd1*^-/-^ mice compared to *Gpr116*^*-/-*^;*Eltd1*^+/+^, *Gpr116*^*-/-*^;*Eltd1*^+/-^, *Gpr116*^*+/+*^;*Eltd1*^-/-^,*Gpr116*^+/-^;*Eltd1*^-/-^ and wild-type mice (*Gpr116*^+/+^;*Eltd1*^+/+^) at 2 and 4 weeks of age. (*, P≤0.05; **, P≤0.01; ***, P≤0.001). (**C**) A representative image of a *Gpr116*^*-/-*^;*Eltd1*^-/-^ mouse compared with a control littermate (*Gpr116*^+/-^;*Eltd1*^-/-^) at 4 weeks of age. (**D**) Genotype of offspring from intercrosses of *Gpr116*^*-/-*^*;Eltd1*^*+/-*^ and *Gpr116*^*+/-*^*;Eltd1*^*-/-*^ mice at E18.5 and P0, respectively.

In order to study the functions of both receptors *in vivo*, mice with global and conditional deficiency of *Gpr116* and *Eltd1* were generated (S3 and S4 Figs). As described before, we did not observe any obvious phenotypical abnormalities in *Gpr116*^*-/-*^ and *Eltd1*^*-/-*^ mice except a defect in lung surfactant homeostasis in *Gpr116*^*-/-*^ mice [12, 14–17]. Given the strong overlap in expression of *Gpr116* and *Eltd1* in endothelial cells, we generated *Gpr116/Eltd1* double deficient (dKO) mice by crossing *Gpr116*^-/-^ animals with *Eltd1*^-/-^ animals. In the offspring of *Gpr116*^*-/-*^*;Eltd1*^*-/+*^ or *Gpr116*^*-/+*^*;Eltd1*^*-/-*^ mice we found significantly reduced number of *Gpr116*^*-/-*^*;Eltd1*^*-/-*^ mice than expected. Double-deficient mice showed a reduced life expectancy with half of the animals surviving the first postnatal month and none of the animals surviving the third postnatal month (Fig 4A). To further analyze the time point of early death of *Gpr116*^*-/-*^*;Eltd1*^*-/-*^ mice, we set up timed matings and analyzed litters before birth (E18.5) as well as after birth (P0). As shown in Fig 4D, normal ratios of mice lacking both *Gpr116* and *Eltd1* were seen alive at embryonic day 18.5; however, half of the *Gpr116*^*-/-*^*;Eltd1*^*-/-*^ mice were found dead shortly after birth. This indicates that about 50% of mice lacking both *Gpr116* and *Eltd1* died perinatally (Fig 4A). The surviving 50% developed significant growth impairment (Fig 4B and 4C) and started to die from the third week after birth. More than 80% of *Gpr116*^*-/-*^*;Eltd1*^*-/-*^ mice had died at 8 weeks of age (Fig 4A).

### Cardiovascular defects in Gpr116/Eltd1 double deficient mice

**Fig 5:**
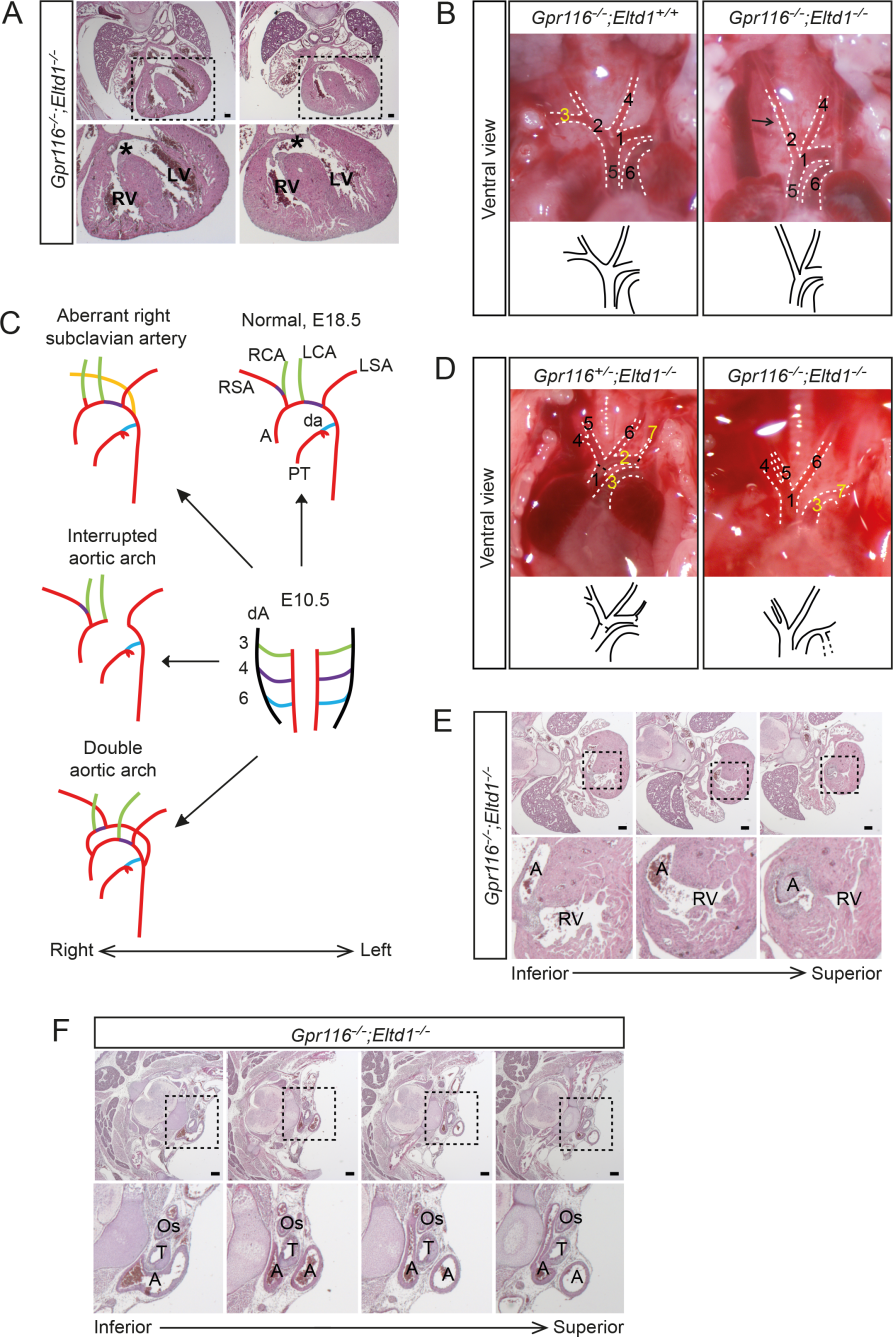
Defects in large arteries and the cardiac outflow tract in GPR116-ELTD1 double deficient embryos (E18.5). (**A**) H&E staining of thoracic sections from two individual *Gpr116*^*-/-*^*;Eltd1*^*-/-*^ embryonic mice with enlarged views of the areas marked by dashed lines shown below. Asterisks indicate the position of the ventricle septum defect. LV, left ventricle; RV, right ventricle. (**B**) Ventral view of large vessels in a *Gpr116*^*-/-*^;*Eltd1*^-/-^ embryonic mouse and a control littermate (*Gpr116*^*-/-*^;*Eltd1*^+/+^) with large arteries marked by dashed lines. A schematic of arteries is shown below. Arrow points to the position where the right subclavian artery usually branches off. 1, Aortic arch; 2, Innominate artery; 3, Right subclavian artery; 4, Left common carotid artery; 5, Ascending thoracic aorta; 6, pulmonary artery. (**C**) Schematic of the embryonic remodeling process of the branchial arch arteries into the aortic arch and the great vessels in normal configuration (E10.5 and E18.5) as well as in disease examples showing aberrant right subclavian artery, interrupted aortic arch and double aortic arch. RSA and LSA, right and left subclavian artery; RCA and LCA, right and left common carotid artery; A, aorta; da, ductus arteriosus; PT, pulmonary trunk. (**D**) Representative ventral view of large vessels from a *Gpr116*^*-/-*^;*Eltd1*^-/-^ embryonic mouse and a control littermate (*Gpr116*^*+/-*^;*Eltd1*^-/-^) with arteries indicated by dashed lines. A schematic of arteries is shown below. 1. Ascending aorta; 2. Aortic arch; 3. Pulmonary artery; 4. Right subclavian artery; 5. Right common carotid artery; 6. Left common carotid artery; 7. Left subclavian artery. 2, 3 and 7 are highlighted in yellow indicating interrupted aortic arch. (**E**) H&E staining of thoracic cross sections (from inferior (left) to superior (right)) from a *Gpr116*^*-/-*^;*Eltd1*^-/-^ embryonic mouse with enlarged views of the dashed line areas shown below. The aorta is connected to the right ventricle, which leads to double outlet right ventricle (DORV). LV, left ventricle; RV, right ventricle. (**F**) H&E staining of thoracic cross sections (from inferior (left) to superior (right)) from a *Gpr116*^*-/-*^;*Eltd1*^-/-^ embryonic mouse with enlarged views of the areas indicated by a dash line shown below, exhibiting a double aortic arch with a retro-esophageal segment. Os, oesophagus; T, trachea; A, aorta. Scale bars: 200 μm (**A, E, F**).

To understand the reason of the perinatal death of dKO newborns, E18.5 dKO embryos and their littermates were dissected and analyzed. In 21 out of 30 analyzed *Gpr116/Eltd1* dKO embryos, we could identify various obvious defects in the heart and the large vessels, whereas Gpr116 and Eltd1 single deficient embryos were indistinguishable from wild-type embryos (Table 1). Among the most frequently observed malformations were ventricle septum defects (VSDs) (Fig 5A). In about 20% of E18.5 dKO mice, the right subclavian artery originating from the innominate artery was missing (Fig 5B). As reported before in other mouse mutants [29, 30], the right subclavian artery instead originated from the descending aorta (Fig 5C and S5 Fig). About 10% of the dKO embryos showed interrupted aortic archs (Fig 5C and 5D), double outlet right ventricles (DORVs) (Fig 5E) or double aortic archs (Fig 5C and 5F). Several of the malformations found at E18.5 were likely to result in perinatal death.

**Table 1:** Frequencies of malformation observed in *Gpr116*^*-/-*^*;Eltd1*^*-/-*^, *Gpr116*^*+/+*^*;Eltd1*^*-/-*^ and *Gpr116*^*-/-*^*;Eltd1*^*+/+*^ E18.5 embryos.

Malformation Genotype	VSD	Abnormal right A. subclavia	Double aortic arch	DORV	Interrupted aortic arch
*Gpr116*^*-/-*^*;Eltd1*^*-/-*^	8/30	6/30	2/30	3/30	2/30
*Gpr116*^*+/+*^*;Eltd1*^*-/-*^	1/10	0/10	0/10	0/10	0/10
*Gpr116*^*-/-*^*;Eltd1*^*+/+*^	0/10	1/10	0/10	0/10	0/10

**Fig 6:**
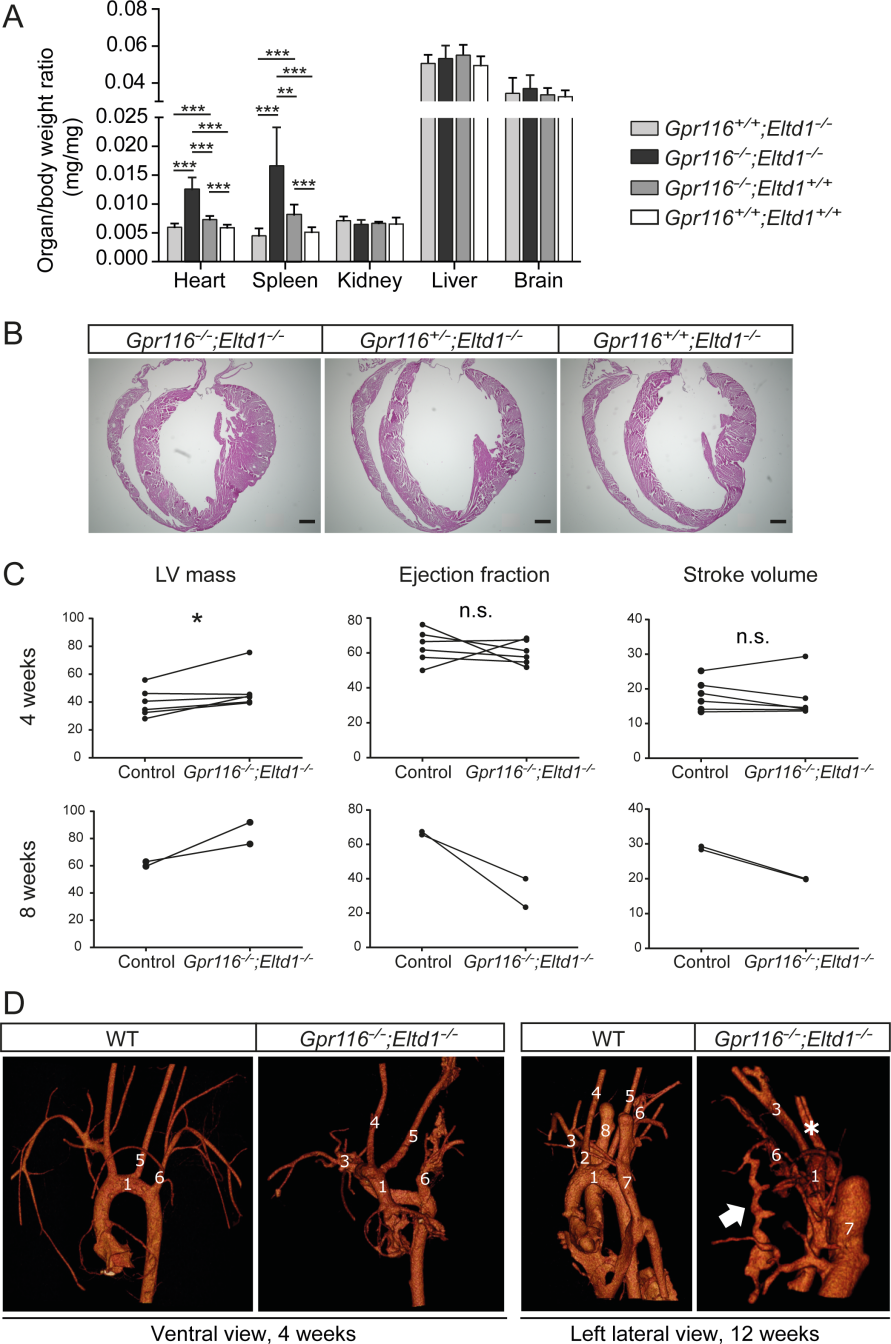
Cardiac hypertrophy, vascular malformations and splenomegaly in 4–12 weeks old GPR116-ELTD1 double deficient mice. (**A**) Organ to body weight ratios in *Gpr116*^*-/-*^;*Eltd1*^-/-^ mice compared to *Gpr116*^*-/-*^;*Eltd1*^+/+^, *Gpr116*^*+/+*^; *Eltd1*^-/-^ and wild-type mice at 4 weeks of age (n = 8 per group; *, P≤0.05; **, P≤0.01; ***, P≤0.001). (**B**) Representative H&E staining of hearts from a *Gpr116*^*-/-*^;*Eltd1*^-/-^ mouse compared to control mice (*Gpr116*^*+/-*^;*Eltd1*^-/-^ and *Gpr11*^*+/+*^;*Eltd1*^-/-^) at 4 weeks of age. (**C**) Cardiac MRI analysis of *Gpr116*^*-/-*^;*Eltd1*^-/-^ mice compared to littermate control mice (*Gpr116*^*+/+*^;*Eltd1*^-/-^ or *Gpr116*^*+/-*^;*Eltd1*^-/-^) at 4 and 8 weeks of age, respectively (*, P≤0.05; n.s., not significant.) (see also S1 and S2 Movies). (**D**) Micro-CT scanning of a vascular corrosion cast from WT and *Gpr116*^*-/-*^;*Eltd1*^-/-^ mice at 4 weeks (left panels) and 12 weeks (right panels) showing the large vessels. Asterisk indicates a common outlet of left and right carotid artery. Arrow indicates a fistula between the left subclavian artery and the left superior vena cava branch. 1. Aortic arch; 2. Innominate artery; 3. Right subclavian artery; 4. Right common carotid artery; 5. Left common carotid artery; 6. Left subclavian artery; 7. Left superior vena cava; 8. Right superior vena cava (see also S3–S6 Movies).

To see whether *Gpr116/Eltd1*-dKO mice survived the perinatal period also had malformations of the cardiovascular system, we analyzed 4-week-old *Gpr116/Eltd1* dKO mice. These animals showed increased relative spleen and heart weights (Fig 6A). Histological analysis revealed a concentric cardiac hypertrophy in dKO mice, which at the same time showed a decreased heart and body size (Fig 6B and S1 and S2 Movies). Magnetic resonance imaging (MRI) analysis of hearts in 4- and 8-week-old mice confirmed an increase in left ventricular (LV) mass (Fig 6C). While the stroke volume and ejection fraction were not significantly different between dKO and littermate control mice at 4 weeks of age, dKO mice exhibited not only a further increase in LV mass, but also severely reduced stroke volume and ejection fraction compared to littermate control animals at 8 weeks of age (Fig 6C and S1 and S2 Movies), indicating myocardial hypertrophy. Micro-CT scanning of vascular corrosion casts from 4 and 12 week old wild-type and dKO mice showed frequently malformations of the large arteries in *Gpr116/Eltd1*-dKO animals including right-sided aortic arch with aberrant left subclavian artery (3 of 18 mice; Fig 6D and S3 and S4 Movies), aberrant right subclavian artery (5 of 18 mice) and a common outlet of left and right carotid arteries (4 of 18 mice) sometimes combined with an arteriovenous fistula between the left subclavian artery and the left vena cava superior branch (2 of 18 mice; Fig 6D and S5 and S6 Movies).

Given that both Gpr116 and Eltd1 are highly expressed in endothelial cells, we wondered whether the phenotype observed in global double deficient mice is due to Gpr116/Eltd1 deficiency in endothelial cells. We therefore crossed *Gpr116*^flox/flox^;*Eltd1*^flox/flox^ with mice expressing Cre under the control of the vascular endothelial cadherin (VE-Cad) promoter (VE-Cadherin-Cre) to generate endothelial cell-specific dKO mice. Endothelial cells were isolated from the lung from endothelial cell-specific dKO mice and control mice). Real-time qPCR was performed to identify expression levels of *Gpr116* and *Eltd1*. Endothelial cell-specific dKO mice showed almost 90% reduction in endothelial levels of RNAs encoding Gpr116 and Eltd1 compared with *Gpr116*^flox/flox^;*Eltd1*^flox/flox^ control mice (S6 Fig). Surprisingly, VE-Cad-Cre;*Gpr116*^flox/flox^;*Eltd1*^flox/flox^ mice survived after birth and showed normal growth, heart and spleen mass when compared with control littermates (*Gpr116*^flox/flox^;*Eltd1*^flox/flox^) (S6 Fig) indicating that Gpr116/Eltd1 deficiency in endothelial cells is not responsible for the phenotypes observed in global dKO mice.

Cardiac neural crest cells also contribute to cardiac outflow tract septation and aortic arch artery patterning [31–33]. To elucidate whether the phenotype observed in global dKO mice is due to Gpr116/Eltd1 deficiency in neural crest cells, we crossed the *Gpr116*^flox/flox^;*Eltd1*^flox/flox^ mice with Pax3-Cre mice [25] to generate neural crest cell-specific dKO mice. However, Pax3-Cre;*Gpr116*^flox/flox^;*Eltd1*^flox/flox^ mice showed no abnormalities compared with control littermates (*Gpr116*^flox/flox^;*Eltd1*^flox/flox^) (S6 Fig).

### Hemolysis and kidney defects in mice lacking Eltd1 and Gpr116

**Fig 7:**
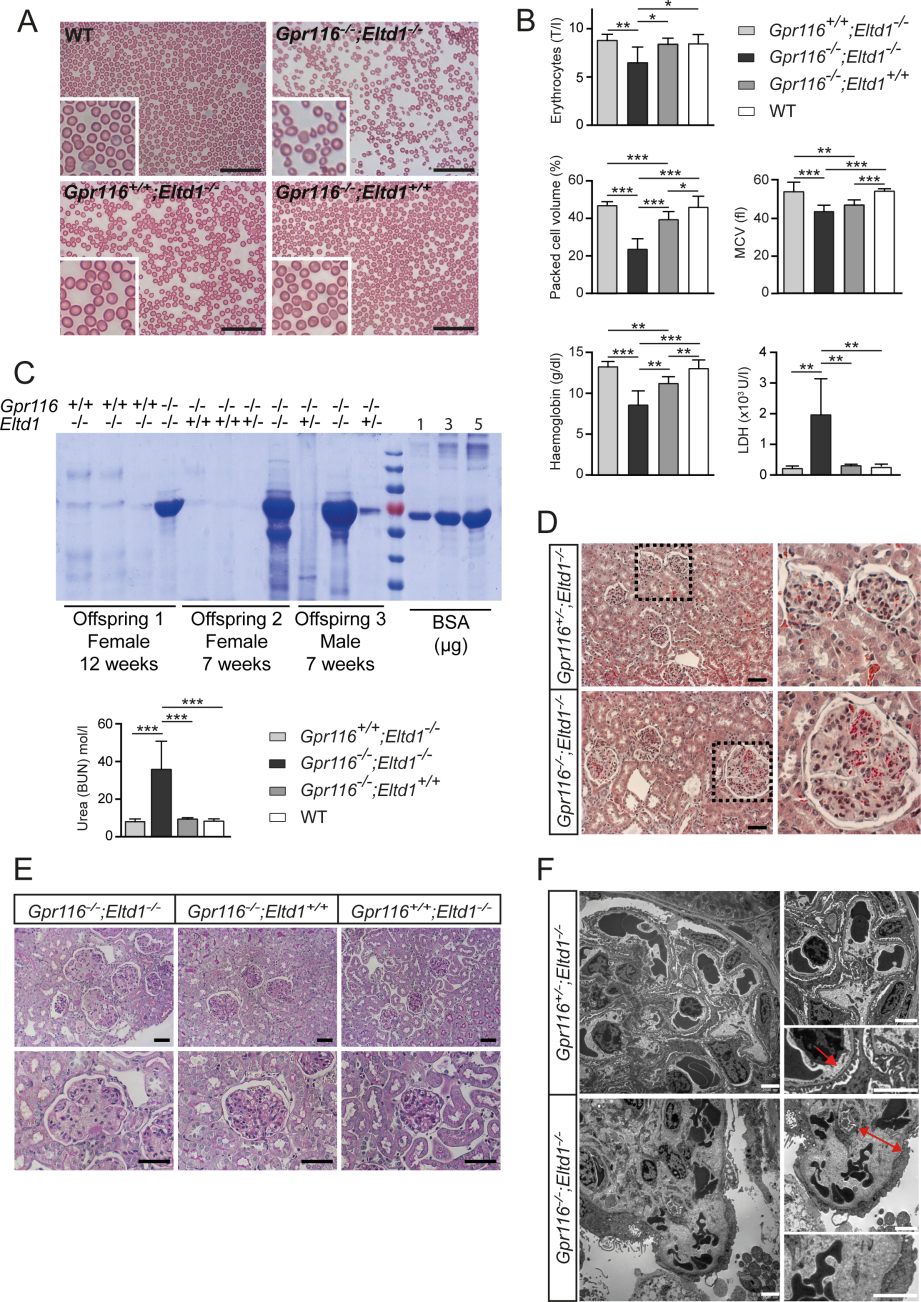
GPR116-ELTD1 double deficient mice developed glomerular thrombotic microangiopathy. (**A**) Giemsa staining of blood smear from dKO mice and controls (insets show enlarged images). (**B**) Analyses of erythrocyte numbers, packed cell volumes and mean corpuscular volumes (MCV) as well as hemoglobin and plasma lactate dehydrogenase (LDH) levels in *Gpr116*^*-/-*^;*Eltd1*^-/-^ mice compared to WT, *Gpr116*^*-/-*^;*Eltd1*^+/+^ and *Gpr116*^*+/+*^;*Eltd1*^-/-^ mice at 4 weeks of age. (n = 8 per group; *, P≤0.05; **, P≤0.01; ***, P≤0.001). (**C**) Upper panel: Protein content of urine from *Gpr116*^*-/-*^;*Eltd1*^-/-^ mice and control littermates (*Gpr116*^*+/+*^*;Eltd1*^*-/-*^, *Gpr116*^*-/-*^*;Eltd1*^*+/+*^, *Gpr116*^*-/-*^*;Eltd1*^*+/-*^) at 7 and 12 weeks of age analyzed by Coomassie Brilliant Blue stain of urinary protein separated by SDS-PAGE. Bovine serum albumin (BSA) is used as standard. Lower panel: Plasma urea concentration in dKO mice and controls at 4 weeks of age. (**D**) Goldner staining of renal glomeruli sections from *Gpr116*^*-/-*^;*Eltd1*^-/-^ and control (*Gpr116*^+/-^;*Eltd1*^-/-^) mice at 4 weeks of age. (**E**) PAS-staining of renal glomeruli sections from *Gpr116*^*-/-*^;*Eltd1*^-/-^ and control (*Gpr116*^*-/-*^;*Eltd1*^+/+^ and *Gpr116*^*+/+*^;*Eltd1*^-/-^) mice at 4 weeks of age. (**F**) Representative transmission electron micrographs of renal glomeruli from *Gpr116*^*-/-*^;*Eltd1*^-/-^ and control (*Gpr116*^*+/-*^;*Eltd1*^-/-^) mice at 4 weeks of age. Arrow indicates the glomerular filtration barrier in the healthy control littermate. Double arrow indicates the expanded subendothelial zone. Scale bars: 50 μm (**A, D, E**); 2500 nm (**F**).

Upon hematological analysis of mice lacking *Eltd1* and *Gpr116*, we observed a large number of misshapen erythrocytes of which many were identified as fragmentocytes (Fig 7A). Erythrocytes numbers, packed cell volume as well as mean corpuscular volume (MCV) were significantly reduced in dKO mice compared to *Gpr116* and *Eltd1* single deficient mice or wild-type animals (Fig 7B). The occurrence of fragmentocytes was accompanied by reduced hemoglobin and increased lactate dehydrogenase (LDH) plasma levels (Fig 7B) indicating increased hemolysis. In addition to changes in erythrocyte size and shape, we observed massive amounts of protein in the urine of mice lacking Gpr116 and Eltd1, but not in littermate controls. In addition, plasma urea levels were significantly elevated in dKO animals indicating kidney failure (Fig 7C). To identify the cause of protein leakage in the kidney, histological and ultrastructural analyses of kidneys from 4-week-old mice were performed. Light microscopy showed massive red blood cell fragments being entrapped in glomerular capillary lumen (Fig 7D) and varying degrees of mesangial matrix expansion (Fig 7E). Transmission electron microscopy (EM) revealed an accumulation of fragmented red blood cells (schistocytes) in the expanded subendothelial zone, decreased capillary lumen and a damaged glomerular filtration barrier including loss of endothelial fenestration and fusion of podocytes foot processes (Fig 7F). The observed microscopic defects of the kidney indicated glomerular thrombotic microangiopathy. Interestingly, no renal defects were observed in mice with constitutive endothelium-specific deficiency of *Gpr116* and *Eltd1* (S6 Fig), indicating that loss of *Gpr116* and/or *Eltd1* in endothelial cells is not responsible for the glomerulopathy observed in global dKO mice. When we analyzed urine of newborn mice, we observed a minor albuminuria in dKO pups compared to Gpr116 and Eltd1 single deficient animals (S7 Fig), whereas no pathological lesions after PAS-staining or upon ultrastructural analysis could be seen at that stage in kidneys of dKO animals (S7 Fig). This indicates that a functional glomerulopathy was already present in dKO mice at birth.

## Discussion

Adhesion GPCRs are a large group of transmembrane proteins whose functions are in most cases still poorly understood. Studies describing their cellular role are often limited since most receptors lack a defined ligand and downstream signaling mechanism [3]. Another approach which has helped to describe functions of adhesion GPCRs is the generation of mice lacking particular members of this receptor group [6]. GPR116 and ELTD1 are two adhesion GPCRs from different families. Previous studies as well as our own expression analysis demonstrated that both receptors are highly expressed in the microvascular endothelium of multiple organs [19], yet Gpr116 or Eltd1 single deficient mice do not show a basic vascular phenotype except a moderate vascular leakage in the brain of animals lacking Gpr116 [17]. This raised the question whether Gpr116 and Eltd1 have redundant vascular functions. In mice lacking Gpr116 and Eltd1 we observed increased perinatal and postnatal lethality. This was accompanied by various cardiovascular malformations occurring with variable penetrance and involving defects in the septation of the cardiac outflow tract such as ventricular septum defects and double outlet right ventricle and defects in the development and/or remodeling of pharyngeal arch arteries including doubled aortic arch, interrupted aortic arch and aberrant right subclavian artery.

These complex morphogenetic processes of pharyngeal arch arteries remodeling and cardiac outflow tract septation requires coordinated communications between cells of mesoderm, endoderm, surface ectoderm, and the neural crest [31, 34, 35]. Defects in the development and/or remodeling of pharyngeal arch arteries result in various malformations such as an interrupted aortic arch, a right-sided aortic arch and an aberrant origin of the right subclavian artery in humans [36]. In mouse models, formation and remodeling of pharyngeal arch arteries has been shown to involve multiple signaling molecules including integrins, endothelins, the transforming growth factor β superfamily, Notch receptors and the Hrt1/Hey1 transcription factor [29, 37–41].

Among these signaling molecules, α5 integrin appears to be of particular interest as its ablation in the anterior mesoderm resulting in abnormalities very similar to those observed in Gpr116/Eltd1 deficient mice including ventricular septum defects and aortic arch malformations [41]. The anterior mesoderm gives rise to all of the cardiac and many of the vascular and muscle lineages in the anterior portion of the embryo, affected also differentiation of neural crest-derived cell into VSMCs in the 4^th^ and 6^th^ pharyngeal arch [41]. A very similar phenotype was observed in mice lacking α5 and αv integrin, but not α5 integrin alone, in the endothelium indicating that α5 integrin expressed by endothelial cells can also contribute to the differentiation of neural crest cells and the development of the cardiac outflow tract and aortic arch vessels [38]. In our study, deletion of Gpr116/Eltd1 in neural crest cells using *Pax3*-Cre knock-in mice exhibited no cardiovascular development abnormalities suggesting that the observed phenotypes in dKOs were not due to functions of Gpr116 and Eltd1 in neural crest cells. The similarity of the cardiovascular developmental defects in α5 integrin mutant and Gpr116/Eltd1 deficient mice may suggest a functional link between these transmembrane proteins. Although α5ß1 integrin has been identified as ligand for another adhesion GPCR, CD97, [42] which shares with Eltd1 the presence of an EGF and a calcium-binding EGF domain in their N-terminal part [3], no interaction between Gpr116 or Eltd1 and integrins has been reported so far. Since neural crest cell and endothelial cell specific loss of Gpr116 and Eltd1 did not result in the phenotype observed in global Gpr116/Eltd1-deficient mice, it remains unclear in which cells function of the two receptors is required for normal formation of large arteries and cardiac outflow tract. It is possible that during development Gpr116 and Eltd1 are expressed in particular cells during defined periods of time which have escaped our expression analysis. Alternatively, both receptors may be expressed in different cell types. It is also conceivable that a small fraction of endothelial cells, in which Gpr116/Eltd1 function is critical, did not undergo recombination in VE-cadherin-Cre transgenic mice.

We have observed a strong increase in cardiac weight in Gpr116/Eltd1-deficient mice already shortly after birth, which was much more pronounced than the increase in heart weight observed in Gpr116 single deficient animals [17]. We think it is most likely that the cardiac hypertrophy in mice lacking both receptors is due to altered hemodynamics caused by defects in the structure of large arteries and the cardiac outflow tract. The hemolysis in addition may contribute to an increased cardiac load. Since none of these defects have been reported in Gpr116 mice, it remains unclear to what degree the cardiac phenotypes of Gpr116 and Gpr116/Eltd1 deficient mice share a common mechanism.

Global *Gpr116/Eltd1* dKO mice developed renal thrombotic microangiopathy, which is indicated by loss of regular glomerular endothelial phenotype with loss of fenestrae, detachment from basement membrane and enlarged subendothelial zones with accumulated schistocytes and damaged glomerular filtration barrier [43]. In addition, dKO mice exhibited fragmented red blood cells, decreased hemoglobin and elevated plasma LDH levels indicating hemolysis. Taken together, these findings are typical clinical and pathological indications of an atypical hemolytic uremic syndrome, which is a form of thrombotic microangiopathy and manifests predominately in the kidney [43]. The atypical hemolytic uremic syndrome is caused by genetic or acquired factors that lead to uncontrolled activation of the alternative complement pathway on the endothelial surface, which results in endothelial cell injury and consequently thrombus formation especially in the kidney [44]. Endothelial damage of capillary vessels can generate abnormally high levels of shear stress, which may be responsible for the fragmentation and hemolysis of erythrocytes [44]. While newborn Gpr116/Eltd1 deficient mice showed no obvious pathological lesions in their kidneys, they already had minor albuminuria indicating a glomerular filtration barrier dysfunction. After birth, dKO mouse showed rapid development of renal thrombotic microangiopathy with severe glomerulopathy at 4 weeks of age. Mutations in several genes, most of them encoding regulatory proteins involved in complement signaling, have been shown to cause atypical hemolytic uremic syndrome [45, 46]. In addition, deficiency of diacylglycerol kinase ɛ has been suggested to lead to excessive arachidonic acid-containing diacylglycerol, which through activation of protein kinase C exert potent pro-thrombotic effects in endothelial cells and platelets, leading to thrombotic microangiopathy [47]. Hence, atypical hemolytic uremic syndrome can be caused by uncontrolled complement pathway or alteration of the anti-thrombogenic properties of the endothelium. Interestingly, no renal defects were observed in mice with constitutive endothelium-specific deficiency of Gpr116 and Eltd1. Therefore, it is reasonable to speculate that Gpr116 and Eltd1 play a role in an unknown cell type related to regulation of the complement system.

We report here about the unexpected finding that loss of the adhesion GPCRs GPR116 and ELTD1 results in malformation of the cardiac outflow tract and the aortic arch arteries as well in the postnatal development of a thrombotic microangiopathy. This indicates that both receptors function together in the development and homeostasis of the vascular system. This work also provides the foundation for exploring the functions of Gpr116 and Eltd1 in the development and remodeling of the cardiac outflow tract and large arteries as well as their potential role in the development of thrombotic microangiopathies. A better understanding of the cell-type specific functions of both receptors and the identification of receptor ligands and downstream signaling mechanisms will be critical for further progress in understanding the role Gpr116 and Eltd1 play in vascular development and function.

## Supporting information

S1 Checklist NC3Rs arrive guidelines checklist. (DOCX)

S1 Fig Schematic structure of the BAC-based GPR116 expression reporter transgene. A bacterial artificial chromosome (BAC) carrying the *GPR116* gene is modified by RecE/RecT-recombineering to insert a cassette consisting of the cDNA encoding the red fluorescent protein mCherry together with the polyadenylation (poly A) signal into the ATG of the *GPR116* coding sequence. Shown are the endogenous locus of the *GPR116* gene (top) and a scheme of the BAC transgene.
(TIF)

S2 Fig Expression of GPR116 and ELTD1 at mouse embryonic day 18.5. Representative fluorescent images (of 3 images per organ from 3 examined animals) of *Gpr116*-mCherry reporter mice (**A**) and *Eltd1*^*lacZ/+*^ mice (**B**) in the heart, lung and skeletal muscle at E18.5. Activity of ß-galactosidase is detected by SPiDER-ßGal, endothelial cells are stained with anti-CD31. Nuclei are counterstained with DAPI. Scale bars: 50 μm.
(TIF)

S3 Fig Generation of Gpr116-floxed and Gpr116-null mice. (**A**) Exon 8 of *Gpr116* was flanked by loxP sites, and a neomycin resistance cassette (NeoR) flanked by FRT-sites was introduced between the two loxP sites. (**B**) After homologous recombination, targeted ES clones were identified by Southern blotting. The BamHI digested ES cell DNA was separated, blotted and hybridized with probe 1. The 12 kb band indicates the wild-type allele and the 10.5 kb band indicates the homologously recombined allele. (**C**) The same ES cell DNA was digested by KpnI and analyzed with probe 2 which detects an 8.4 kb band for the wild-type allele and a 10.2 kb band for the homologously recombined allele. (**D**) Expression of flp recombinase recombined the FRT sites and resulted in deletion of the neomycin selection cassette, leading to a *Gpr116* floxed allele; Subsequent expression of Cre-recombinase recombined the loxP sites and resulted in deletion of the exon 8, leading to a *Gpr116* null allele. (**E**) The genotype of *Gpr116*-floxed animals was analyzed by PCR using primers 1 and 2 leading to a 287 bp product for the wild-type allele and 375 bp for the floxed allele. (**F**) The genotype of *Gpr116*-null animals was analyzed by PCR using primers 1, 3 and 4 resulting in a 352 bp product for the wild-type allele and 267 bp for the null allele. (**G**) Deletion of exon 8 in *Gpr116* mRNA was confirmed by RT-PCR using primers 5 and 6 resulted in a 310 bp product from wild-type mice and a 164 bp product from *Gpr116*^-/-^ mice.
(TIF)

S4 Fig Generation of Eltd1-floxed and Eltd1-null mice. (**A**) Exon 5 of *Eltd1* was flanked by loxP sites, and a neomycin resistance cassette (NeoR) flanked by FRT-sites was introduced between the two loxP sites. (**B**) After homologous recombination, targeted ES clones were identified by Southern blotting. The EcoRV-digested ES cell DNA was separated, blotted and hybridized with probe 1. The 11.1 kb band indicates the wild-type allele with a C57B6 background (and <11.1 kb with a SV129 background) and the 12.9 kb band indicates the homologously recombined allele. WT^a^, ES cell hybrid with 50% C57B6 and 50% SV129 backgrounds; WT^b^, ES cell with a pure SV129 background. (**C**) The same ES cell DNA was digested by HindIII and analyzed with probe 2 which detects an 8.2 kb band for the wild-type allele and a 10 kb band for the homologous recombined allele. (**D**) Expression of flp recombinase recombined FRT sites and resulted after deletion of the neomycin selection cassette in a floxed allele. Subsequent expression of Cre-recombinase recombined the loxP sites and resulted in the deletion of the exon 5, creating an *Eltd1* null allele. (**E**) The genotype of *Eltd1*-floxed animals was analyzed by PCR using primers 1 and 2 which produced a 239 bp product for the wild-type allele and 390 bp for the floxed allele. (**F**) The genotype of *Eltd1*-null animals was analyzed by PCR using primers 1, 3 and 4, giving a product of 189 bpt for the wild-type allele and of 302 bp for the null allele. (**G**) Deletion of exon 5 in *Eltd1* mRNA was confirmed by RT-PCR using primers 5 and 6 giving a 481 bp product from wild-type mice, and a 410 bp product for *Eltd1*^*-/-*^ mice.
(TIF)

S5 Fig Representative histological analysis of large arteries in a mouse lacking GPR116 and ELTD1 with an aberrant right subclavian artery. Example of a *Gpr116*^-/-^;*Eltd1*^-/-^ mouse at E18.5 with an aberrant right subclavian artery (red star) which originates from the descending aorta (DA) and then crosses to the right side behind the esophagus (E) and the trachea (T). AA, aortic arch; DAB, ductus arteriosus Botalli; ASCA, ascending aorta.
(TIF)

S6 Fig Analysis of endothelial cell- and neural crest cell-specific GPR116-ELTD1 double deficient mice. (**A**) mRNA expression levels of *Gpr116* and *Eltd1* in FACS-purified wildtype, *Gpr116*^flox/flox^;*Eltd1*^flox/flox^ and VE-Cad-Cre;*Gpr116*^flox/flox^;*Eltd1*^flox/flox^ endothelial cells identified by real-time qPCR (mean ± SD, n = 3 per group). (**B**) Body weight, organ to body weight ratios of heart and spleen of VE-Cad-Cre;*Gpr116*^flox/flox^;*Eltd1*^flox/flox^ and control (*Gpr116*^flox/flox^;*Eltd1*^flox/flox^) mice at 6 weeks of age (n = 3, n.s. not significant). (**C**) Urinary protein of VE-Cad-Cre;*Gpr116*^flox/flox^;*Eltd1*^flox/flox^ and control mice at 9 and 22 weeks of age. Urinary protein was detected by Coomassie Brilliant Blue on a SDS-PAGE gel with BSA as standard. (**D**) Body weight, organ to body weight ratios of heart and spleen of Pax3-Cre;*Gpr116*^flox/flox^;*Eltd1*^flox/flox^ and control (*Gpr116*^flox/flox^;*Eltd1*^flox/flox^) mice at 14 weeks of age (n = 4, n.s. not significant).
(TIF)

S7 Fig Analysis of the kidney in GPR116-ELTD1 double deficient mice at P0. (**A**) Urinary protein of *Gpr116*^*-/-*^;*Eltd1*^-/-^ mice and control littermates (*Gpr116*^*-/-*^;*Eltd1*^+/+^ and *Gpr116*^*+/+*^;*Eltd1*^-/-^) shortly after birth (P0) is shown by Coomassie Brilliant Blue of a SDS-PAGE gel. Bovine serum albumin (BSA) was used as standard. (**B**) Representative transmission electron micrographs of renal glomeruli from *Gpr116*^*-/-*^;*Eltd1*^-/-^ and control (*Gpr116*^*+/-*^;*Eltd1*^-/-^) mice. (**C**) PAS-staining of renal glomeruli sections from *Gpr116*^*-/-*^;*Eltd1*^-/-^ and control (*Gpr116*^*-/-*^;*Eltd1*^+/+^ and *Gpr116*^*+/+*^;*Eltd1*^-/-^) mice. Scale bars: 2500 nm (**B**); 50 μm (**C**).
(TIF)

S1 Movie Cardiac MRI of 8 weeks old littermate control. (MPG)

S2 Movie Cardiac MRI of 8 weeks old dKO mouse. (MPG)

S3 Movie Micro-CT analysis of a vascular corrosion cast from 4 weeks old wild type control. (MOV)

S4 Movie Micro-CT analysis of a vascular corrosion cast from 4 weeks old dKO mouse. (MOV)

S5 Movie Micro-CT analysis of a vascular corrosion cast from 12 weeks old wild-type control. (MOV)

S6 Movie Micro-CT analysis of a vascular corrosion cast from 12 weeks old dKO mouse. (MOV)

## References

[1] Bjarnadottir TK, Gloriam DE, Hellstrand SH, Kristiansson H, Fredriksson R, Schioth HB. Comprehensive repertoire and phylogenetic analysis of the G protein-coupled receptors in human and mouse. Genomics. 2006;88(3):263–73. doi: 10.1016/j.ygeno.2006.04.001 . 16753280 10.1016/j.ygeno.2006.04.001

[2] Bjarnadottir TK, Fredriksson R, Hoglund PJ, Gloriam DE, Lagerstrom MC, Schioth HB. The human and mouse repertoire of the adhesion family of G-protein-coupled receptors. Genomics. 2004;84(1):23–33. doi: 10.1016/j.ygeno.2003.12.004 . 15203201 10.1016/j.ygeno.2003.12.004

[3] Hamann J, Aust G, Arac D, Engel FB, Formstone C, Fredriksson R, et al International Union of Basic and Clinical Pharmacology. XCIV. Adhesion G protein-coupled receptors. Pharmacol Rev. 2015;67(2):338–67. doi: 10.1124/pr.114.009647 ; PubMed Central PMCID: PMCPMC4394687. 25713288 10.1124/pr.114.009647 PMC4394687

[4] Lin HH, Stacey M, Yona S, Chang GW. GPS proteolytic cleavage of adhesion-GPCRs. Adv Exp Med Biol. 2010;706:49–58. . 21618825 10.1007/978-1-4419-7913-1_4

[5] Arac D, Boucard AA, Bolliger MF, Nguyen J, Soltis SM, Sudhof TC, et al A novel evolutionarily conserved domain of cell-adhesion GPCRs mediates autoproteolysis. EMBO J. 2012;31(6):1364–78. doi: 10.1038/emboj.2012.26 ; PubMed Central PMCID: PMC3321182. 22333914 10.1038/emboj.2012.26 PMC3321182

[6] Langenhan T, Aust G, Hamann J. Sticky signaling—adhesion class G protein-coupled receptors take the stage. Sci Signal. 2013;6(276):re3 doi: 10.1126/scisignal.2003825 . 23695165 10.1126/scisignal.2003825

[7] Paavola KJ, Stephenson JR, Ritter SL, Alter SP, Hall RA. The N terminus of the adhesion G protein-coupled receptor GPR56 controls receptor signaling activity. J Biol Chem. 2011;286(33):28914–21. doi: 10.1074/jbc.M111.247973 ; PubMed Central PMCID: PMC3190698. 21708946 10.1074/jbc.M111.247973 PMC3190698

[8] Liebscher I, Schon J, Petersen SC, Fischer L, Auerbach N, Demberg LM, et al A tethered agonist within the ectodomain activates the adhesion G protein-coupled receptors GPR126 and GPR133. Cell Rep. 2014;9(6):2018–26. doi: 10.1016/j.celrep.2014.11.036 ; PubMed Central PMCID: PMCPMC4277498. 25533341 10.1016/j.celrep.2014.11.036 PMC4277498

[9] Stoveken HM, Hajduczok AG, Xu L, Tall GG. Adhesion G protein-coupled receptors are activated by exposure of a cryptic tethered agonist. Proc Natl Acad Sci U S A. 2015;112(19):6194–9. doi: 10.1073/pnas.1421785112 ; PubMed Central PMCID: PMC4434738. 25918380 10.1073/pnas.1421785112 PMC4434738

[10] Favara DM, Banham AH, Harris AL. A review of ELTD1, a pro-angiogenic adhesion GPCR. Biochem Soc Trans. 2014;42(6):1658–64. doi: 10.1042/BST20140216 . 25399586 10.1042/BST20140216

[11] Nechiporuk T, Urness LD, Keating MT. ETL, a novel seven-transmembrane receptor that is developmentally regulated in the heart. ETL is a member of the secretin family and belongs to the epidermal growth factor-seven-transmembrane subfamily. J Biol Chem. 2001;276(6):4150–7. doi: 10.1074/jbc.M004814200 . 11050079 10.1074/jbc.M004814200

[12] Xiao J, Jiang H, Zhang R, Fan G, Zhang Y, Jiang D, et al Augmented cardiac hypertrophy in response to pressure overload in mice lacking ELTD1. PLoS One. 2012;7(5):e35779 doi: 10.1371/journal.pone.0035779 ; PubMed Central PMCID: PMC3350503. 22606234 10.1371/journal.pone.0035779 PMC3350503

[13] Masiero M, Simoes FC, Han HD, Snell C, Peterkin T, Bridges E, et al A core human primary tumor angiogenesis signature identifies the endothelial orphan receptor ELTD1 as a key regulator of angiogenesis. Cancer Cell. 2013;24(2):229–41. doi: 10.1016/j.ccr.2013.06.004 ; PubMed Central PMCID: PMC3743050. 23871637 10.1016/j.ccr.2013.06.004 PMC3743050

[14] Bridges JP, Ludwig MG, Mueller M, Kinzel B, Sato A, Xu Y, et al Orphan G protein-coupled receptor GPR116 regulates pulmonary surfactant pool size. Am J Respir Cell Mol Biol. 2013;49(3):348–57. doi: 10.1165/rcmb.2012-0439OC ; PubMed Central PMCID: PMC3824053. 23590306 10.1165/rcmb.2012-0439OC PMC3824053

[15] Fukuzawa T, Ishida J, Kato A, Ichinose T, Ariestanti DM, Takahashi T, et al Lung surfactant levels are regulated by Ig-Hepta/GPR116 by monitoring surfactant protein D. PLoS One. 2013;8(7):e69451 doi: 10.1371/journal.pone.0069451 ; PubMed Central PMCID: PMC3726689. 23922714 10.1371/journal.pone.0069451 PMC3726689

[16] Yang MY, Hilton MB, Seaman S, Haines DC, Nagashima K, Burks CM, et al Essential regulation of lung surfactant homeostasis by the orphan G protein-coupled receptor GPR116. Cell Rep. 2013;3(5):1457–64. doi: 10.1016/j.celrep.2013.04.019 ; PubMed Central PMCID: PMC3695742. 23684610 10.1016/j.celrep.2013.04.019 PMC3695742

[17] Niaudet C, Hofmann JJ, Mae MA, Jung B, Gaengel K, Vanlandewijck M, et al Gpr116 Receptor Regulates Distinctive Functions in Pneumocytes and Vascular Endothelium. PLoS One. 2015;10(9):e0137949 doi: 10.1371/journal.pone.0137949 ; PubMed Central PMCID: PMCPMC4579087. 26394398 10.1371/journal.pone.0137949 PMC4579087

[18] Nie T, Hui X, Gao X, Li K, Lin W, Xiang X, et al Adipose tissue deletion of Gpr116 impairs insulin sensitivity through modulation of adipose function. FEBS Lett. 2012;586(20):3618–25. doi: 10.1016/j.febslet.2012.08.006 . 22971422 10.1016/j.febslet.2012.08.006

[19] Wallgard E, Larsson E, He L, Hellstrom M, Armulik A, Nisancioglu MH, et al Identification of a core set of 58 gene transcripts with broad and specific expression in the microvasculature. Arterioscler Thromb Vasc Biol. 2008;28(8):1469–76. doi: 10.1161/ATVBAHA.108.165738 . 18483404 10.1161/ATVBAHA.108.165738

[20] Takase H, Matsumoto K, Yamadera R, Kubota Y, Otsu A, Suzuki R, et al Genome-wide identification of endothelial cell-enriched genes in the mouse embryo. Blood. 2012;120(4):914–23. doi: 10.1182/blood-2011-12-398156 . 22535667 10.1182/blood-2011-12-398156

[21] Dieterich LC, Mellberg S, Langenkamp E, Zhang L, Zieba A, Salomaki H, et al Transcriptional profiling of human glioblastoma vessels indicates a key role of VEGF-A and TGFbeta2 in vascular abnormalization. J Pathol. 2012;228(3):378–90. doi: 10.1002/path.4072 . 22786655 10.1002/path.4072

[22] Rodriguez CI, Buchholz F, Galloway J, Sequerra R, Kasper J, Ayala R, et al High-efficiency deleter mice show that FLPe is an alternative to Cre-loxP. Nat Genet. 2000;25(2):139–40. doi: 10.1038/75973 . 10835623 10.1038/75973

[23] Lakso M, Pichel JG, Gorman JR, Sauer B, Okamoto Y, Lee E, et al Efficient in vivo manipulation of mouse genomic sequences at the zygote stage. Proc Natl Acad Sci U S A. 1996;93(12):5860–5. . 8650183 10.1073/pnas.93.12.5860 PMC39152

[24] Alva JA, Zovein AC, Monvoisin A, Murphy T, Salazar A, Harvey NL, et al VE-Cadherin-Cre-recombinase transgenic mouse: a tool for lineage analysis and gene deletion in endothelial cells. Dev Dyn. 2006;235(3):759–67. doi: 10.1002/dvdy.20643 . 16450386 10.1002/dvdy.20643

[25] Engleka KA, Gitler AD, Zhang M, Zhou DD, High FA, Epstein JA. Insertion of Cre into the Pax3 locus creates a new allele of Splotch and identifies unexpected Pax3 derivatives. Dev Biol. 2005;280(2):396–406. doi: 10.1016/j.ydbio.2005.02.002 . 15882581 10.1016/j.ydbio.2005.02.002

[26] Jennemann R, Rothermel U, Wang S, Sandhoff R, Kaden S, Out R, et al Hepatic glycosphingolipid deficiency and liver function in mice. Hepatology. 2010;51(5):1799–809. doi: 10.1002/hep.23545 . 20432257 10.1002/hep.23545

[27] Kaur H, Takefuji M, Ngai CY, Carvalho J, Bayer J, Wietelmann A, et al Targeted Ablation of Periostin-Expressing Activated Fibroblasts Prevents Adverse Cardiac Remodeling in Mice. Circ Res. 2016;118(12):1906–17. doi: 10.1161/CIRCRESAHA.116.308643 . 27140435 10.1161/CIRCRESAHA.116.308643

[28] Kaur H, Carvalho J, Looso M, Singh P, Chennupati R, Preussner J, et al Single-cell profiling reveals heterogeneity and functional patterning of GPCR expression in the vascular system. Nat Commun. 2017;8:15700 doi: 10.1038/ncomms15700 . 28621310 10.1038/ncomms15700 PMC5481776

[29] Kurihara Y, Kurihara H, Oda H, Maemura K, Nagai R, Ishikawa T, et al Aortic arch malformations and ventricular septal defect in mice deficient in endothelin-1. J Clin Invest. 1995;96(1):293–300. doi: 10.1172/JCI118033 . 7615798 10.1172/JCI118033 PMC185200

[30] Makki N, Capecchi MR. Cardiovascular defects in a mouse model of HOXA1 syndrome. Hum Mol Genet. 2012;21(1):26–31. doi: 10.1093/hmg/ddr434 ; PubMed Central PMCID: PMCPMC3235008. 21940751 10.1093/hmg/ddr434 PMC3235008

[31] Stoller JZ, Epstein JA. Cardiac neural crest. Semin Cell Dev Biol. 2005;16(6):704–15. doi: 10.1016/j.semcdb.2005.06.004 . 16054405 10.1016/j.semcdb.2005.06.004

[32] Gittenberger-de Groot AC, Bartelings MM, Poelmann RE, Haak MC, Jongbloed MR. Embryology of the heart and its impact on understanding fetal and neonatal heart disease. Semin Fetal Neonatal Med. 2013;18(5):237–44. doi: 10.1016/j.siny.2013.04.008 . 23886508 10.1016/j.siny.2013.04.008

[33] Keyte A, Hutson MR. The neural crest in cardiac congenital anomalies. Differentiation. 2012;84(1):25–40. doi: 10.1016/j.diff.2012.04.005 ; PubMed Central PMCID: PMCPMC3389200. 22595346 10.1016/j.diff.2012.04.005 PMC3389200

[34] Rentschler S, Jain R, Epstein JA. Tissue-tissue interactions during morphogenesis of the outflow tract. Pediatr Cardiol. 2010;31(3):408–13. doi: 10.1007/s00246-009-9611-2 ; PubMed Central PMCID: PMCPMC2951316. 20039033 10.1007/s00246-009-9611-2 PMC2951316

[35] Gittenberger-de Groot AC, Calkoen EE, Poelmann RE, Bartelings MM, Jongbloed MR. Morphogenesis and molecular considerations on congenital cardiac septal defects. Ann Med. 2014;46(8):640–52. doi: 10.3109/07853890.2014.959557 . 25307363 10.3109/07853890.2014.959557

[36] Barry A. The aortic arch derivatives in human adult. Anat Rec. 1951;111(2):221–38. . 14894834 10.1002/ar.1091110207

[37] Fujita M, Sakabe M, Ioka T, Watanabe Y, Kinugasa-Katayama Y, Tsuchihashi T, et al Pharyngeal arch artery defects and lethal malformations of the aortic arch and its branches in mice deficient for the Hrt1/Hey1 transcription factor. Mech Dev. 2016;139:65–73. doi: 10.1016/j.mod.2015.11.002 . 26577899 10.1016/j.mod.2015.11.002

[38] van der Flier A, Badu-Nkansah K, Whittaker CA, Crowley D, Bronson RT, Lacy-Hulbert A, et al Endothelial alpha5 and alphav integrins cooperate in remodeling of the vasculature during development. Development. 2010;137(14):2439–49. doi: 10.1242/dev.049551 ; PubMed Central PMCID: PMCPMC2889609. 20570943 10.1242/dev.049551 PMC2889609

[39] Gittenberger-de Groot AC, Azhar M, Molin DG. Transforming growth factor beta-SMAD2 signaling and aortic arch development. Trends Cardiovasc Med. 2006;16(1):1–6. doi: 10.1016/j.tcm.2005.09.006 . 16387623 10.1016/j.tcm.2005.09.006

[40] High FA, Zhang M, Proweller A, Tu L, Parmacek MS, Pear WS, et al An essential role for Notch in neural crest during cardiovascular development and smooth muscle differentiation. J Clin Invest. 2007;117(2):353–63. doi: 10.1172/JCI30070 ; PubMed Central PMCID: PMCPMC1783803. 17273555 10.1172/JCI30070 PMC1783803

[41] Liang D, Wang X, Mittal A, Dhiman S, Hou SY, Degenhardt K, et al Mesodermal expression of integrin alpha5beta1 regulates neural crest development and cardiovascular morphogenesis. Dev Biol. 2014;395(2):232–44. doi: 10.1016/j.ydbio.2014.09.014 ; PubMed Central PMCID: PMCPMC4252364. 25242040 10.1016/j.ydbio.2014.09.014 PMC4252364

[42] Wang T, Ward Y, Tian L, Lake R, Guedez L, Stetler-Stevenson WG, et al CD97, an adhesion receptor on inflammatory cells, stimulates angiogenesis through binding integrin counterreceptors on endothelial cells. Blood. 2005;105(7):2836–44. doi: 10.1182/blood-2004-07-2878 . 15576472 10.1182/blood-2004-07-2878

[43] Barbour T, Johnson S, Cohney S, Hughes P. Thrombotic microangiopathy and associated renal disorders. Nephrol Dial Transplant. 2012;27(7):2673–85. doi: 10.1093/ndt/gfs279 ; PubMed Central PMCID: PMCPMC3398067. 22802583 10.1093/ndt/gfs279 PMC3398067

[44] Noris M, Mescia F, Remuzzi G. STEC-HUS, atypical HUS and TTP are all diseases of complement activation. Nat Rev Nephrol. 2012;8(11):622–33. doi: 10.1038/nrneph.2012.195 . 22986360 10.1038/nrneph.2012.195

[45] Rodriguez de Cordoba S, Hidalgo MS, Pinto S, Tortajada A. Genetics of atypical hemolytic uremic syndrome (aHUS). Semin Thromb Hemost. 2014;40(4):422–30. doi: 10.1055/s-0034-1375296 . 24799305 10.1055/s-0034-1375296

[46] Kavanagh D, Goodship TH, Richards A. Atypical hemolytic uremic syndrome. Semin Nephrol. 2013;33(6):508–30. doi: 10.1016/j.semnephrol.2013.08.003 ; PubMed Central PMCID: PMCPMC3863953. 24161037 10.1016/j.semnephrol.2013.08.003 PMC3863953

[47] Lemaire M, Fremeaux-Bacchi V, Schaefer F, Choi M, Tang WH, Le Quintrec M, et al Recessive mutations in DGKE cause atypical hemolytic-uremic syndrome. Nat Genet. 2013;45(5):531–6. doi: 10.1038/ng.2590 ; PubMed Central PMCID: PMCPMC3719402. 23542698 10.1038/ng.2590 PMC3719402

