# Supplementary material for: Developmental vascular remodeling defects and postnatal kidney failure in mice lacking Gpr116 (Adgrf5) and Eltd1 (Adgrl4)
Source: PLoS One. 2017 Aug 14;12(8):e0183166. doi: 10.1371/journal.pone.0183166 (PMC5555693; doi:10.1371/journal.pone.0183166)

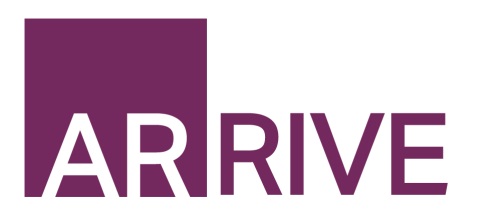


The ARRIVE Guidelines Checklist

Animal Research: Reporting In Vivo Experiments

Carol Kilkenny^1^, William J Browne^2^, Innes C Cuthill^3^, Michael Emerson^4^ and Douglas G Altman^5^

*^1^The National Centre for the Replacement, Refinement and Reduction of Animals in Research, London, UK, ^2^School of Veterinary Science, University of Bristol, Bristol, UK, ^3^School of Biological Sciences, University of Bristol, Bristol, UK, ^4^National Heart and Lung Institute, Imperial College London, UK, ^5^Centre for Statistics in Medicine, University of Oxford, Oxford, UK.*

|  | | ITEM | RECOMMENDATION | Section/ Paragraph |
| --- | --- | --- | --- | --- |
| 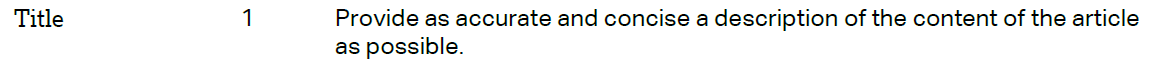 | | | Page 1: Title |  |
| 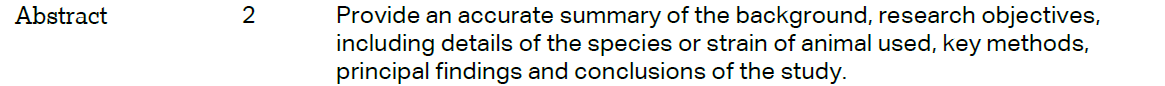 | | | Page 2: Abstract |  |
| INTRODUCTION | | |  |  |
| 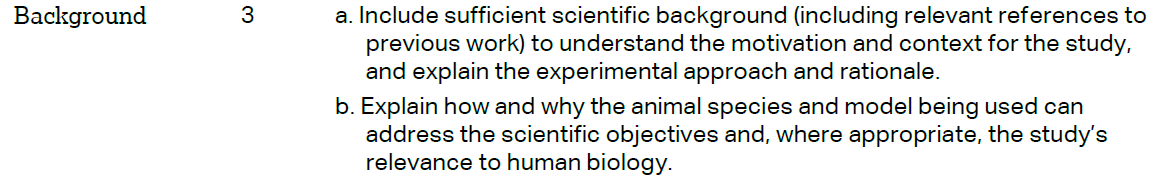 | | | Introduction: §1-2 |  |
| 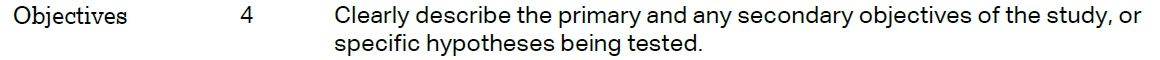 | | | Introduction: §3 |  |
| METHODS | | |  |  |
| 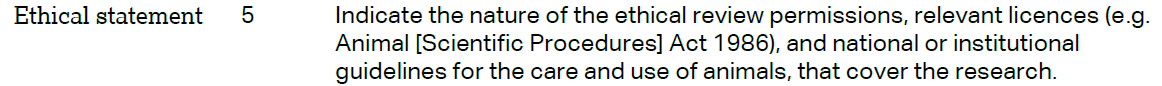 | | | M&M/Animals/§3 |  |
| 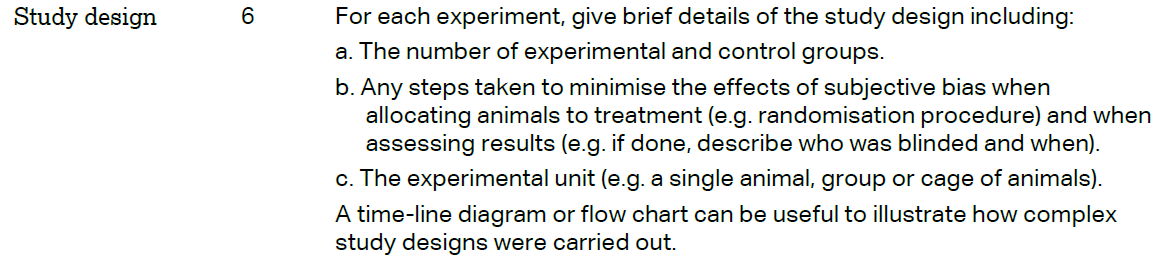 | | |  |  |
| 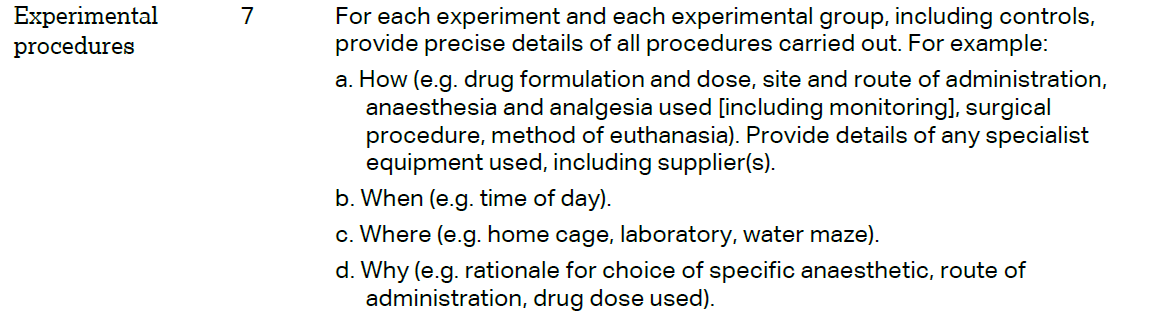 | | | M&M/MRI; Vascular corrosion casting |  |
| 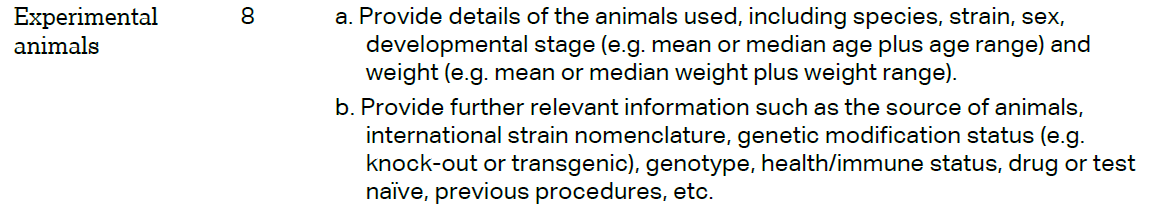 | | | M&M/Animals/§1-2 |  |

The ARRIVE guidelines. Originally published in *PLoS Biology*, June 2010^1^

| 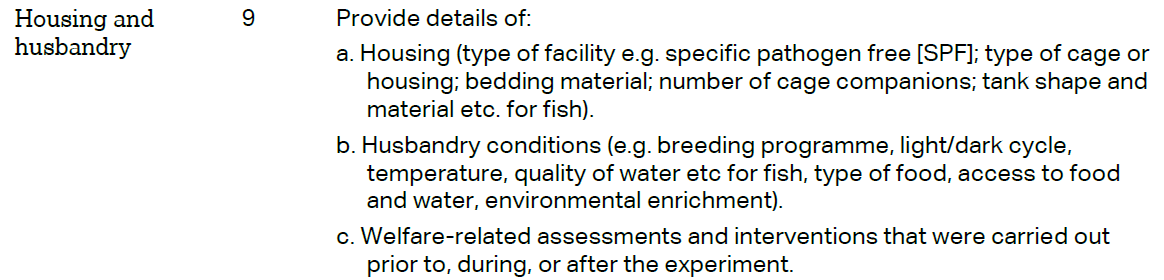 | M&M/Animals/§3 | |
| --- | --- | --- |
| 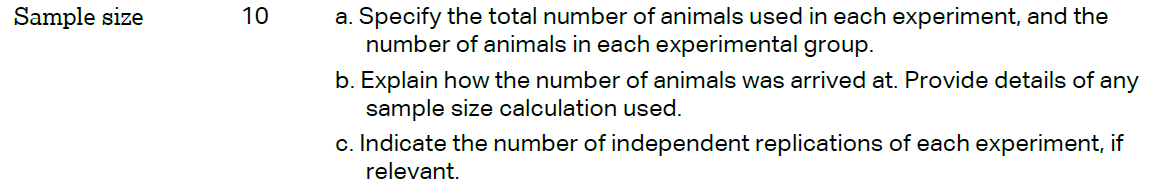 | Results/ Fig 4; Table 1; Fig 6 and Fig 7 | |
| 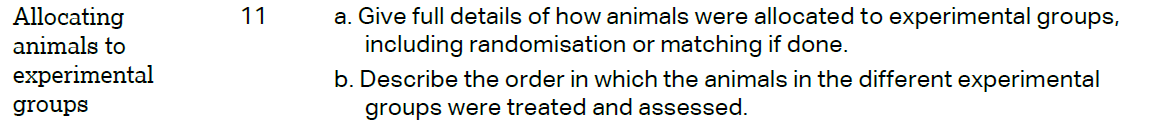 |  | |
| 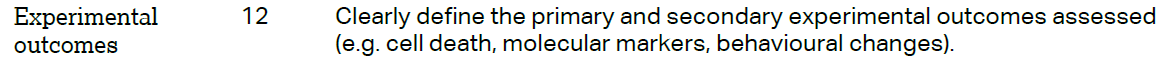 |  | |
| 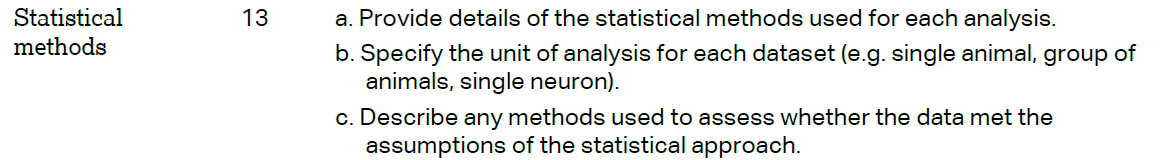 | M&M/Statistics | |
| RESULTS |  | |
| 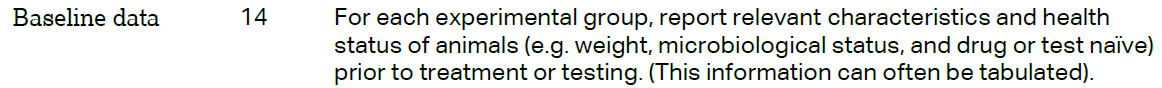 | Results/Fig 4 | |
| 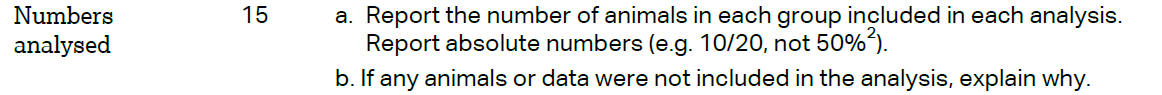 | Results/ Fig 4; Table 1; Fig 6 and Fig 7 | |
| 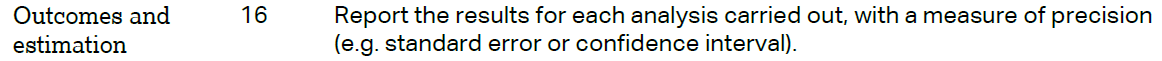 | Results/ Fig 4; Fig 6 and Fig 7 | |
| 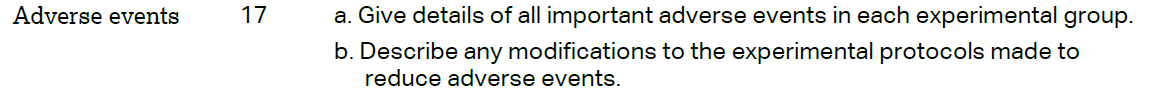 | Results/ Fig 4; Fig 6 and Fig 7 | |
| DISCUSSION |  | |
| 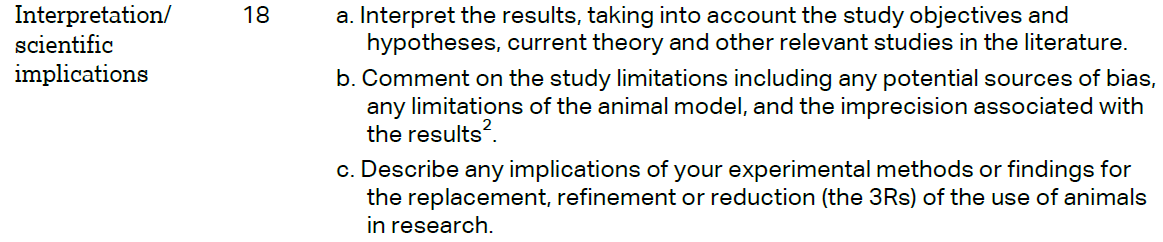 | Discussion/§1-6 | |
| 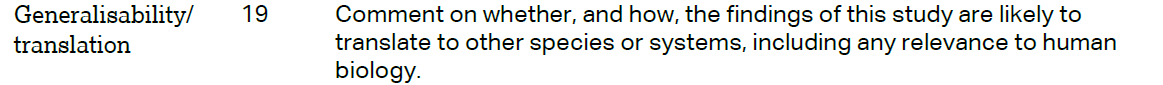 |  | |
| 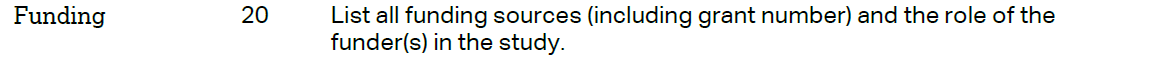 | |  |


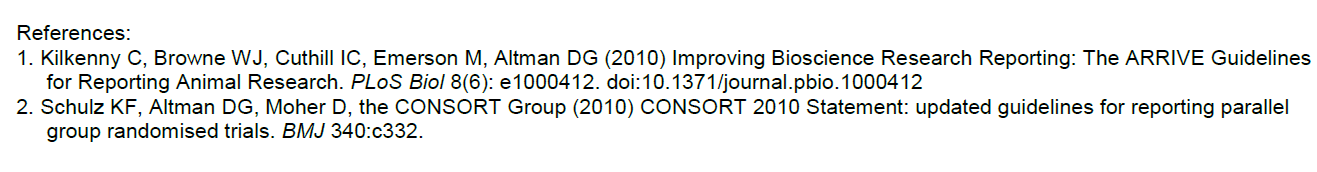

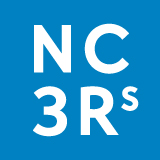

Supplement: S1 Checklist — (DOCX) [file pone.0183166.s001.docx]
