# Supplementary figures and images for: Developmental vascular remodeling defects and postnatal kidney failure in mice lacking Gpr116 (Adgrf5) and Eltd1 (Adgrl4)
Source: PLoS One. 2017 Aug 14;12(8):e0183166. doi: 10.1371/journal.pone.0183166 (PMC5555693; doi:10.1371/journal.pone.0183166)

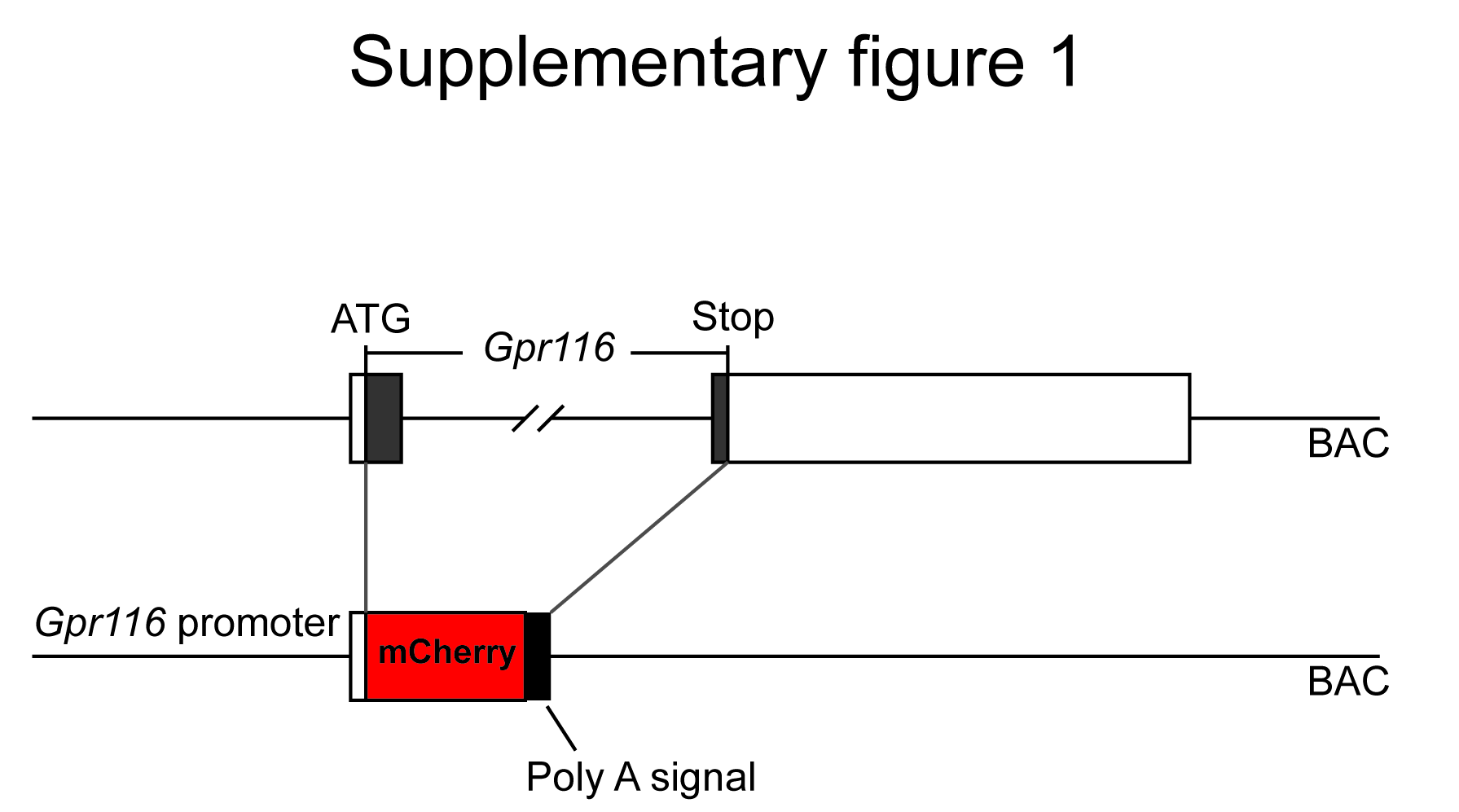

Supplement: S1 Fig — A bacterial artificial chromosome (BAC) carrying the GPR116 gene is modified by RecE/RecT-recombineering to insert a cassette consisting of the cDNA encoding the red fluorescent protein mCherry together with the polyadenylation (poly A) signal into the ATG of the GPR116 coding sequence. Shown are the endogenous locus of the GPR116 gene (top) and a scheme of the BAC transgene. (TIF) [file pone.0183166.s002.tif]

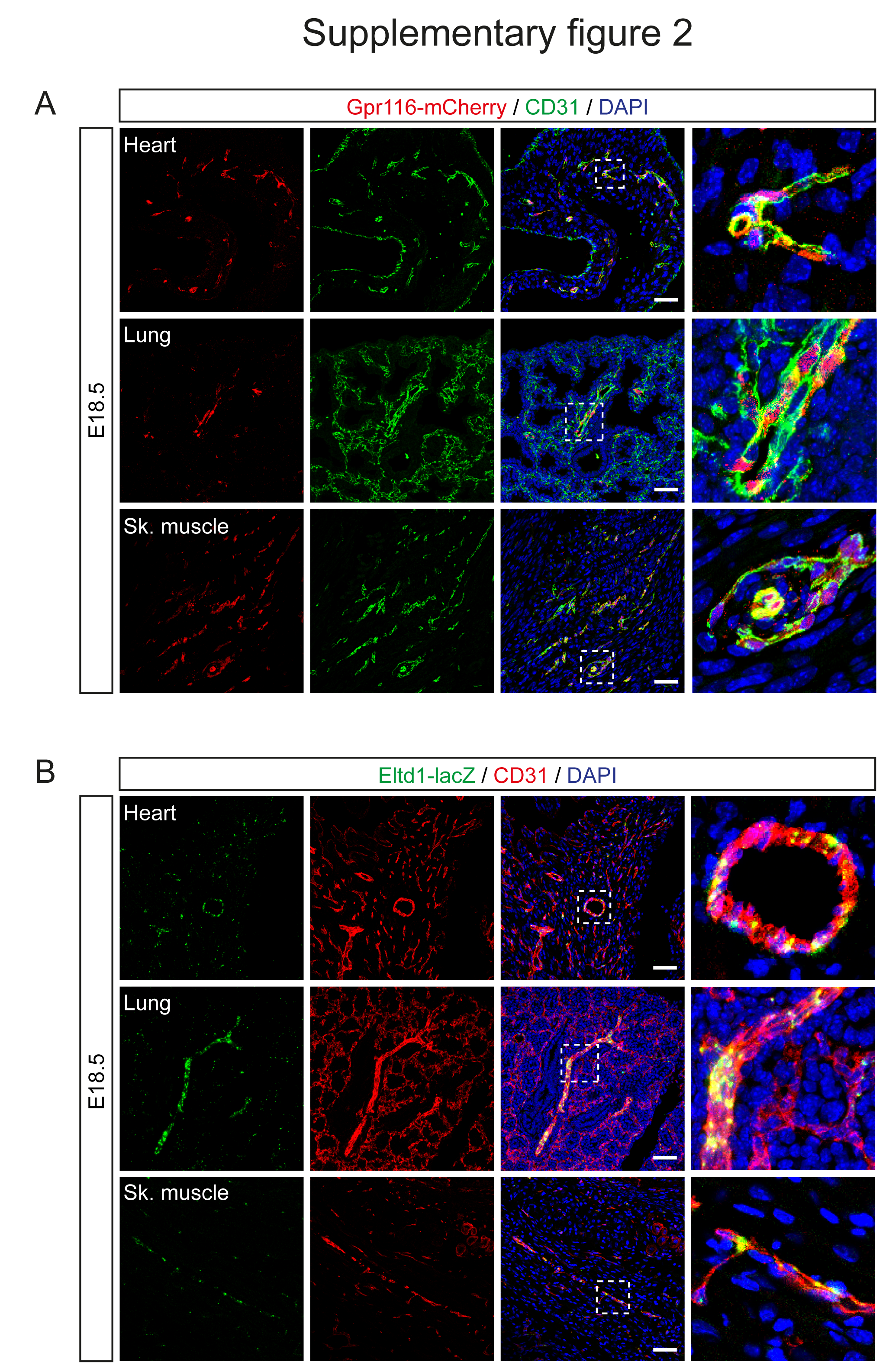

Supplement: S2 Fig — Representative fluorescent images (of 3 images per organ from 3 examined animals) of Gpr116-mCherry reporter mice (A) and Eltd1lacZ/+ mice (B) in the heart, lung and skeletal muscle at E18.5. Activity of ß-galactosidase is detected by SPiDER-ßGal, endothelial cells are stained with anti-CD31. Nuclei are counterstained with DAPI. Scale bars: 50 μm. (TIF) [file pone.0183166.s003.tif]

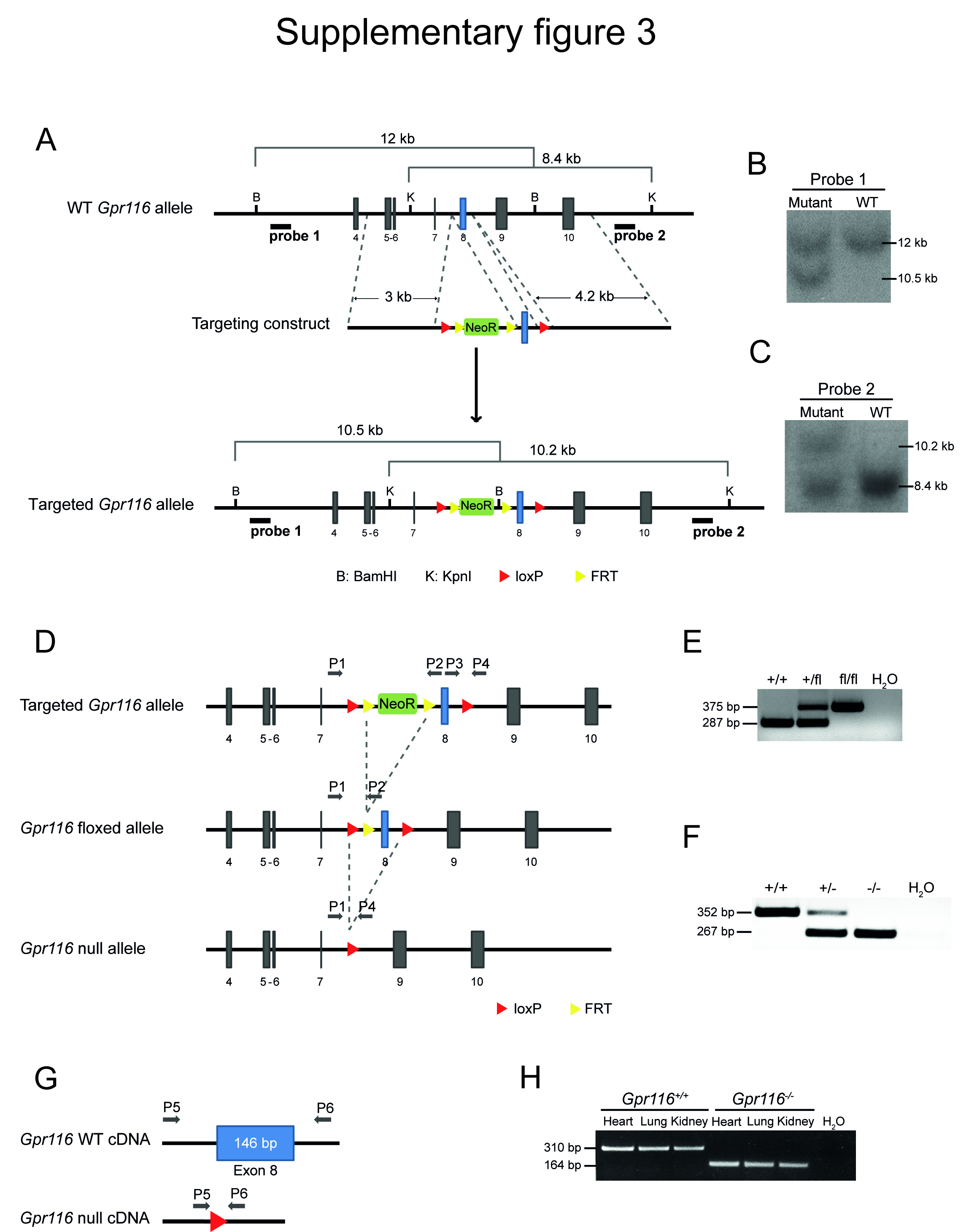

Supplement: S3 Fig — (A) Exon 8 of Gpr116 was flanked by loxP sites, and a neomycin resistance cassette (NeoR) flanked by FRT-sites was introduced between the two loxP sites. (B) After homologous recombination, targeted ES clones were identified by Southern blotting. The BamHI digested ES cell DNA was separated, blotted and hybridized with probe 1. The 12 kb band indicates the wild-type allele and the 10.5 kb band indicates the homologously recombined allele. (C) The same ES cell DNA was digested by KpnI and analyzed with probe 2 which detects an 8.4 kb band for the wild-type allele and a 10.2 kb band for the homologously recombined allele. (D) Expression of flp recombinase recombined the FRT sites and resulted in deletion of the neomycin selection cassette, leading to a Gpr116 floxed allele; Subsequent expression of Cre-recombinase recombined the loxP sites and resulted in deletion of the exon 8, leading to a Gpr116 null allele. (E) The genotype of Gpr116-floxed animals was analyzed by PCR using primers 1 and 2 leading to a 287 bp product for the wild-type allele and 375 bp for the floxed allele. (F) The genotype of Gpr116-null animals was analyzed by PCR using primers 1, 3 and 4 resulting in a 352 bp product for the wild-type allele and 267 bp for the null allele. (G) Deletion of exon 8 in Gpr116 mRNA was confirmed by RT-PCR using primers 5 and 6 resulted in a 310 bp product from wild-type mice and a 164 bp product from Gpr116-/- mice. (TIF) [file pone.0183166.s004.tif]

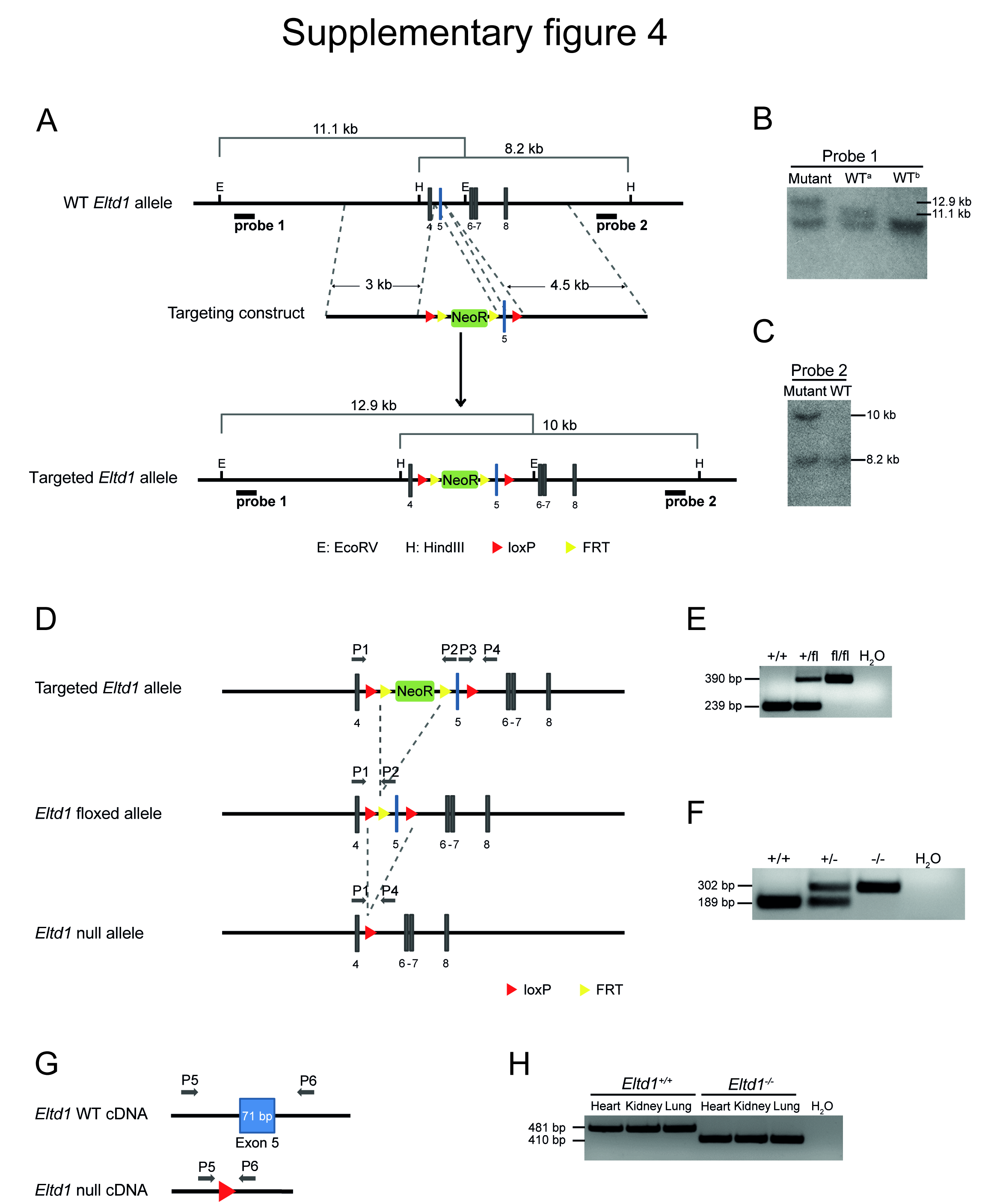

Supplement: S4 Fig — (A) Exon 5 of Eltd1 was flanked by loxP sites, and a neomycin resistance cassette (NeoR) flanked by FRT-sites was introduced between the two loxP sites. (B) After homologous recombination, targeted ES clones were identified by Southern blotting. The EcoRV-digested ES cell DNA was separated, blotted and hybridized with probe 1. The 11.1 kb band indicates the wild-type allele with a C57B6 background (and <11.1 kb with a SV129 background) and the 12.9 kb band indicates the homologously recombined allele. WTa, ES cell hybrid with 50% C57B6 and 50% SV129 backgrounds; WTb, ES cell with a pure SV129 background. (C) The same ES cell DNA was digested by HindIII and analyzed with probe 2 which detects an 8.2 kb band for the wild-type allele and a 10 kb band for the homologous recombined allele. (D) Expression of flp recombinase recombined FRT sites and resulted after deletion of the neomycin selection cassette in a floxed allele. Subsequent expression of Cre-recombinase recombined the loxP sites and resulted in the deletion of the exon 5, creating an Eltd1 null allele. (E) The genotype of Eltd1-floxed animals was analyzed by PCR using primers 1 and 2 which produced a 239 bp product for the wild-type allele and 390 bp for the floxed allele. (F) The genotype of Eltd1-null animals was analyzed by PCR using primers 1, 3 and 4, giving a product of 189 bpt for the wild-type allele and of 302 bp for the null allele. (G) Deletion of exon 5 in Eltd1 mRNA was confirmed by RT-PCR using primers 5 and 6 giving a 481 bp product from wild-type mice, and a 410 bp product for Eltd1-/- mice. (TIF) [file pone.0183166.s005.tif]

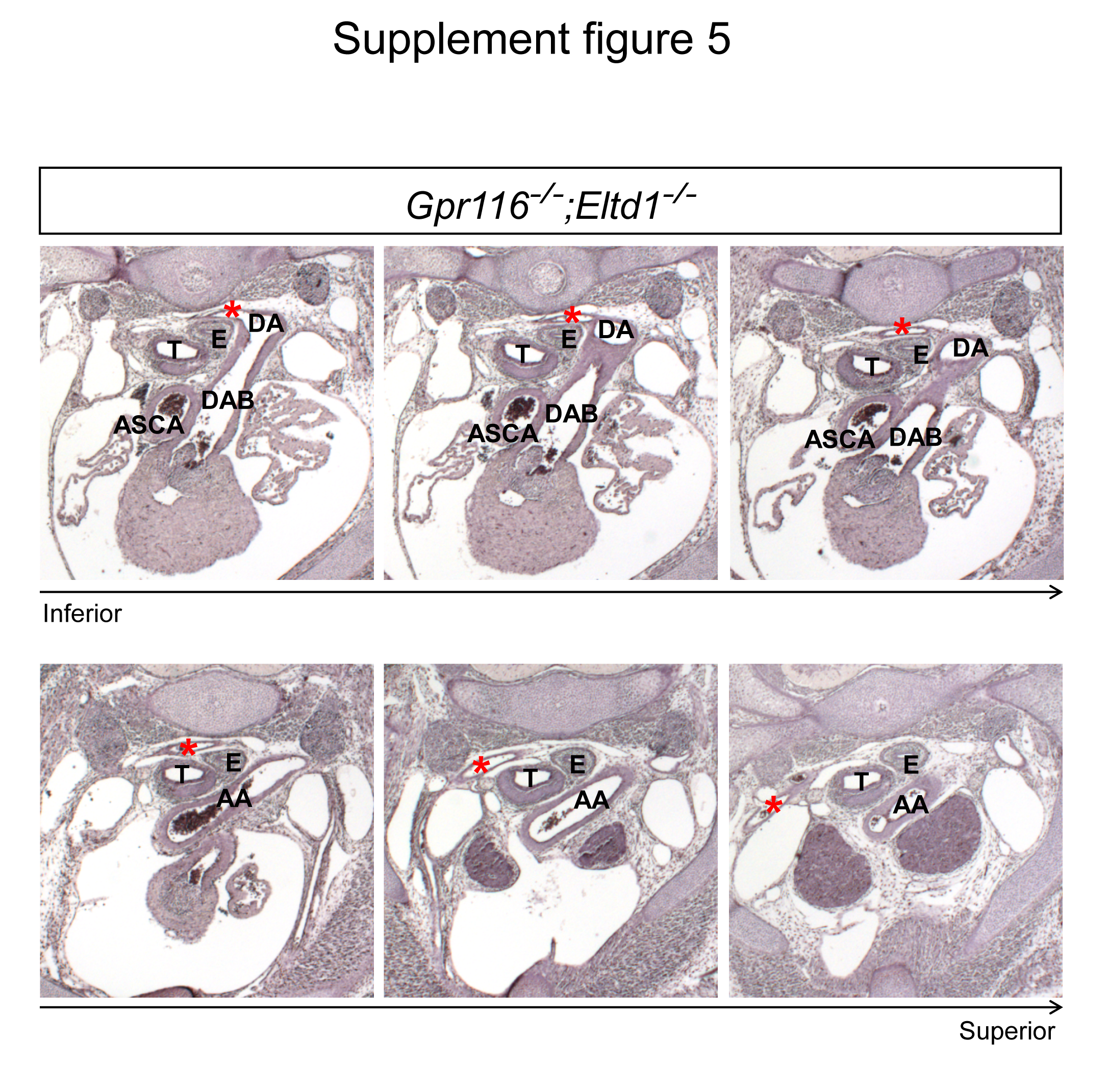

Supplement: S5 Fig — Example of a Gpr116-/-;Eltd1-/- mouse at E18.5 with an aberrant right subclavian artery (red star) which originates from the descending aorta (DA) and then crosses to the right side behind the esophagus (E) and the trachea (T). AA, aortic arch; DAB, ductus arteriosus Botalli; ASCA, ascending aorta. (TIF) [file pone.0183166.s006.tif]

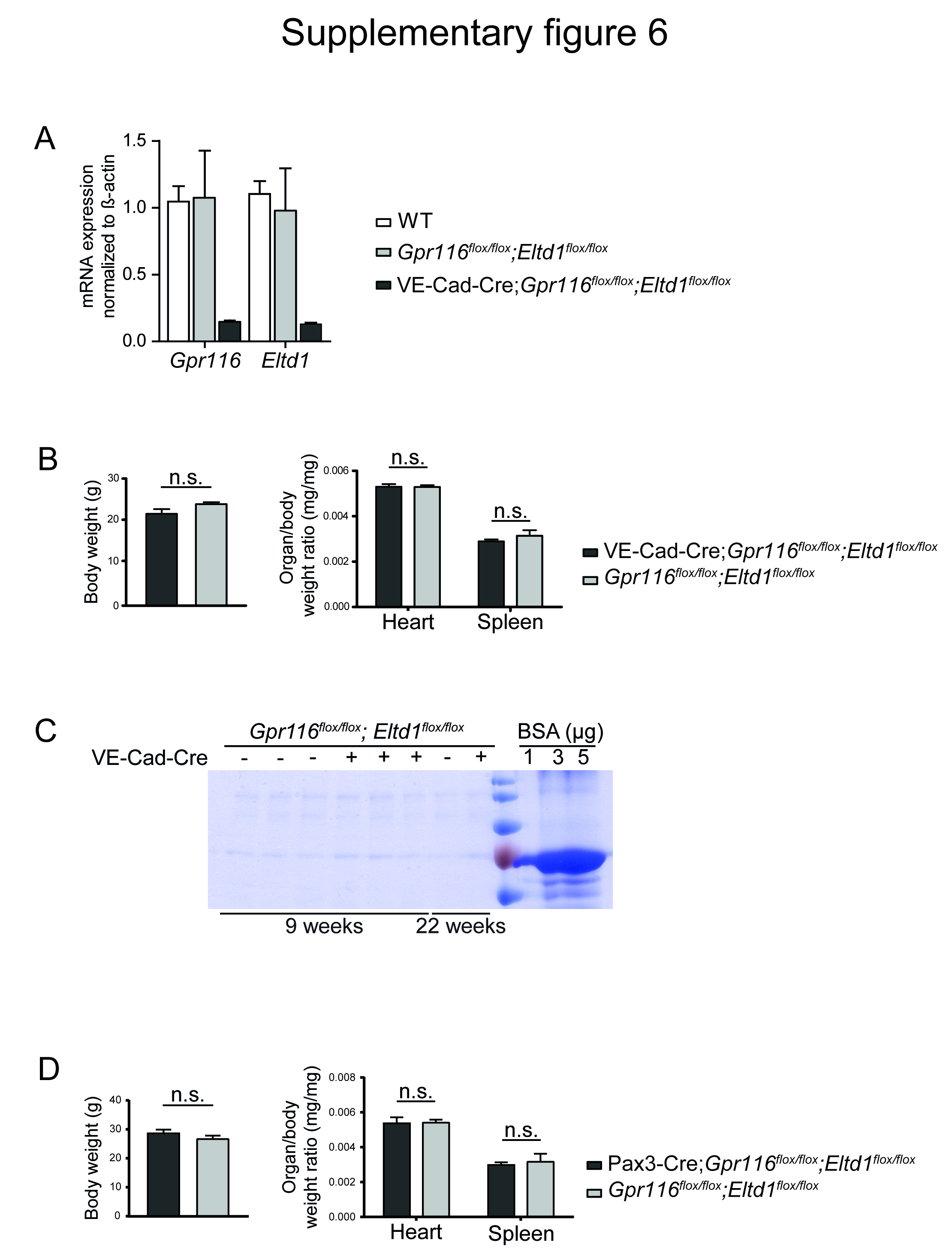

Supplement: S6 Fig — (A) mRNA expression levels of Gpr116 and Eltd1 in FACS-purified wildtype, Gpr116flox/flox;Eltd1flox/flox and VE-Cad-Cre;Gpr116flox/flox;Eltd1flox/flox endothelial cells identified by real-time qPCR (mean ± SD, n = 3 per group). (B) Body weight, organ to body weight ratios of heart and spleen of VE-Cad-Cre;Gpr116flox/flox;Eltd1flox/flox and control (Gpr116flox/flox;Eltd1flox/flox) mice at 6 weeks of age (n = 3, n.s. not significant). (C) Urinary protein of VE-Cad-Cre;Gpr116flox/flox;Eltd1flox/flox and control mice at 9 and 22 weeks of age. Urinary protein was detected by Coomassie Brilliant Blue on a SDS-PAGE gel with BSA as standard. (D) Body weight, organ to body weight ratios of heart and spleen of Pax3-Cre;Gpr116flox/flox;Eltd1flox/flox and control (Gpr116flox/flox;Eltd1flox/flox) mice at 14 weeks of age (n = 4, n.s. not significant). (TIF) [file pone.0183166.s007.tif]

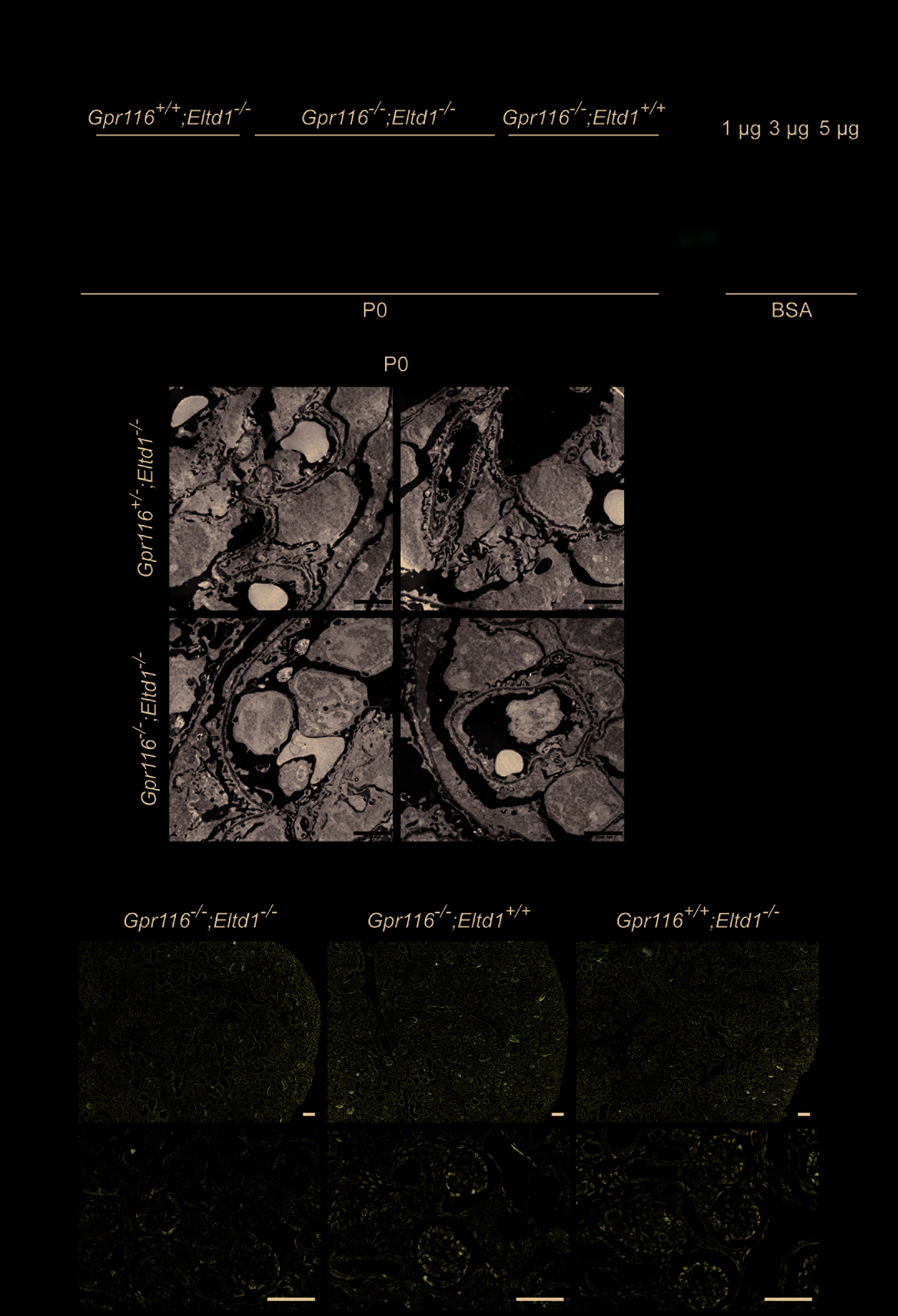

Supplement: S7 Fig — (A) Urinary protein of Gpr116-/-;Eltd1-/- mice and control littermates (Gpr116-/-;Eltd1+/+ and Gpr116+/+;Eltd1-/-) shortly after birth (P0) is shown by Coomassie Brilliant Blue of a SDS-PAGE gel. Bovine serum albumin (BSA) was used as standard. (B) Representative transmission electron micrographs of renal glomeruli from Gpr116-/-;Eltd1-/- and control (Gpr116+/-;Eltd1-/-) mice. (C) PAS-staining of renal glomeruli sections from Gpr116-/-;Eltd1-/- and control (Gpr116-/-;Eltd1+/+ and Gpr116+/+;Eltd1-/-) mice. Scale bars: 2500 nm (B); 50 μm (C). (TIF) [file pone.0183166.s008.tif]
